# Supplementary material for: Cancer cell genetics shaping of the tumor microenvironment reveals myeloid cell-centric exploitable vulnerabilities in hepatocellular carcinoma
Source: Nat Commun. 2024 Mar 22;15:2581. doi: 10.1038/s41467-024-46835-2 (PMC10959959; doi:10.1038/s41467-024-46835-2)
Supplement: Supplementary file 3 — Description of Additional Supplementary Files [file 41467_2024_46835_MOESM3_ESM.pdf]

Description of Additional Supplementary Files

Supplementary Data 1: Bulk Tumor transcriptional signatures from RNAseq for each genetically-distinct HCC model

Supplementary Data 2: TCGA LIHC expression and patients segregated according to HCC murine signatures

Supplementary Data 3: Whole Exome Sequencing analyses of HCC patients and genetically-disntinct HCC murine models

Supplementary Data 4: Clinical information from TMA HCC patients

Supplementary Data 5: CD45+ RNAseq expression for each genetically-distinct HCC model

Supplementary Data 6: ssGSEA Biocarta CD45+ RNAseq

Supplementary Data 7: Populations derived from scRNAseq of NrasG12D and Control liver

Supplementary Data 8: Differential expression in each population derived from scRNAseq comparing NrasG12D relative to Control liver

Supplementary Data 9: Differential expression from cancer cell lines RNAseq with ERK2 inhibitor and TF motif analysis

Supplementary Data 10: IHC antibodies

Supplementary Data 11: Flow Cytometry Panel
