# Supplementary material for: Cancer cell genetics shaping of the tumor microenvironment reveals myeloid cell-centric exploitable vulnerabilities in hepatocellular carcinoma
Source: Nat Commun. 2024 Mar 22;15:2581. doi: 10.1038/s41467-024-46835-2 (PMC10959959; doi:10.1038/s41467-024-46835-2)
Supplement: Supplementary file 15 — Reporting Summary [file 41467_2024_46835_MOESM15_ESM.pdf]

nature portfolio

Corresponding author(s): Dr. Serena Vegna and Dr. Leila Akkari

Last updated by author(s): Mar 11, 2024

Reporting Summary

Nature Portfolio wishes to improve the reproducibility of the work that we publish. This form provides structure for consistency and transparency in reporting. For further information on Nature Portfolio policies, see our Editorial Policies and the Editorial Policy Checklist.

Statistics

For all statistical analyses, confirm that the following items are present in the figure legend, table legend, main text, or Methods section.

n/a	Confirmed	
☐	☒	The exact sample size (*n*) for each experimental group/condition, given as a discrete number and unit of measurement
☐	☒	A statement on whether measurements were taken from distinct samples or whether the same sample was measured repeatedly
☐	☒	The statistical test(s) used AND whether they are one- or two-sided<br>*Only common tests should be described solely by name; describe more complex techniques in the Methods section.*
☐	☒	A description of all covariates tested
☐	☒	A description of any assumptions or corrections, such as tests of normality and adjustment for multiple comparisons
☐	☒	A full description of the statistical parameters including central tendency (e.g. means) or other basic estimates (e.g. regression coefficient) AND variation (e.g. standard deviation) or associated estimates of uncertainty (e.g. confidence intervals)
☐	☒	For null hypothesis testing, the test statistic (e.g. *F*, *t*, *r*) with confidence intervals, effect sizes, degrees of freedom and *P* value noted<br>*Give P values as exact values whenever suitable.*
☒	☐	For Bayesian analysis, information on the choice of priors and Markov chain Monte Carlo settings
☒	☐	For hierarchical and complex designs, identification of the appropriate level for tests and full reporting of outcomes
☐	☒	Estimates of effect sizes (e.g. Cohen's *d*, Pearson's *r*), indicating how they were calculated

Our web collection on statistics for biologists contains articles on many of the points above.

Software and code

Policy information about availability of computer code

Data collection

No software was used.

Data analysis

Data analysis was performed with the following software and tools:
Seqpurge (2022_11)
Hisat2 (2.1.0)
gensum (0.2.1)
NearestTemplatePrediction (v.4 2015-12-02)
GSEA (v.4.2.2)
single sample gene set enrichment analysis (ssGSEA v. 10.0.11)
Metascape (v.3.5.20240101)
Cell Ranger (6.1.2)
Burrows–Wheeler alignment (v.0.5.10)
GATK (v.4.2.6.0)
SNPEff and SnpSift (v.5.1)
Simple Enrichment Analysis (SEA v. 5.4.1) tool from the MEME suite

All the analyses and visualization were performed using the following R (4.2.2, 2022-10-31) packages:
ggplot2(v.3.4.2)

ggpubr (v.0.6.0)
ComplexHeatmap (v.2.14.0)
EdgeR (v.3.40.2).
Hyper (v.1.14.0)
UCSCXenaTools (v.1.4.8)
survival (v.3.4-0)
survminer (v.0.4.9)
Seurat (4.1.3)
MiloR (v.1.6.0)
enrichR (v.3.2)
clusterProfiler (v.4.6.2)

For manuscripts utilizing custom algorithms or software that are central to the research but not yet described in published literature, software must be made available to editors and reviewers. We strongly encourage code deposition in a community repository (e.g. GitHub). See the Nature Portfolio guidelines for submitting code & software for further information.

Data

Policy information about availability of data

All manuscripts must include a data availability statement. This statement should provide the following information, where applicable:

- Accession codes, unique identifiers, or web links for publicly available datasets
- A description of any restrictions on data availability
- For clinical datasets or third party data, please ensure that the statement adheres to our policy

The RNA-seq, scRNA-seq and WES publicly available data generated in this study are available in the Gene Expression Omnibus (GEO) database under accession code GSE216717 (https://www.ncbi.nlm.nih.gov/geo/query/acc.cgi?acc=GSE216717) . The GM-CSF signature was reanalysed from PRJEB9884 (https://www.ebi.ac.uk/ena/browser/view/PRJEB9884). The publicly available human HCC data was sourced from PRJCA007744 (https://ngdc.cncb.ac.cn/bioproject/browse/PRJCA007744). The remaining data are available within the Article, Supplementary Information or Source Data file. Further information and requests for resources and reagents should be directed to the corresponding authors.

Research involving human participants, their data, or biological material

Policy information about studies with human participants or human data. See also policy information about sex, gender (identity/presentation), and sexual orientation and race, ethnicity and racism.

Reporting on sex and gender	An independent cohort of 488 both male (432) and female (56) HCC patients of who underwent primary curative resection between 2006 and 2010 were enrolled.No sex-and gender-based analyses were performed.<br>We also included report on data acquired from TCGA LIHC patients, here no sex-and gender-based analyses were performed.
Reporting on race, ethnicity, or other socially relevant groupings	no variables on race, ethnicity or socially relevant groupings to report
Population characteristics	The clinical characteristics of patient are summarized in Supplemental Data 4.
Recruitment	The inclusion criteria used for patient enrollment from the consecutive cohorts were as follows: no anticancer therapies; no diagnosis or history of any other concurrent malignancies; no concurrent autoimmune diseases, HIV or syphilis; and available followup data. The exclusion criteria were Child-Pugh C liver function and evidence of hepatic decompensation, which included refractory ascites, esophageal or gastric variceal bleeding, or hepatic encephalopathy.
Ethics oversight	For experiments using human samples, all samples were anonymously coded in accordance with the local ethical guidelines (as stipulated by the Declaration of Helsinki). This study was approved by the Institutional Review Boards of the Sun Yat-sen University Cancer Center (Guangzhou, China). Written informed consent was obtained from all patients as stated in the study from Wu, C et al., J Clin Invest. 2020 PMID: 32497024.

Note that full information on the approval of the study protocol must also be provided in the manuscript.

Field-specific reporting

Please select the one below that is the best fit for your research. If you are not sure, read the appropriate sections before making your selection.

☒ Life sciences ☐ Behavioural & social sciences ☐ Ecological, evolutionary & environmental sciences

For a reference copy of the document with all sections, see nature.com/documents/nr-reporting-summary-flat.pdf

Life sciences study design

All studies must disclose on these points even when the disclosure is negative.

Sample size	For a power calculation, the mean and standard deviation were estimated based on previous results. An alpha of 0.05 and power of 0.8 was taken as a guideline in these calculations.
Data exclusions	No data was excluded from the study
Replication	All the in vitro experiments (except for MAPK array) were repeated in at least 3 independent experiments and all attempts at replication were successful. At least 3 Biological replicates were used (except for scRNA seq and cytokine array) to ensure reproducibility. For the MAPK array, we were able to validate the main observed results by wesbernblot in supplementary figure S6a with 4 independent experiment repeats.For cytokine array, we used the results to validate transcriptional candidates that were differentially regulated at bulk tumor level in the Nras models in figure 5c. The main target candidate Csf2 we further validated with GM-CSF ELISA in various independent experiments in figure 5d and 6. For scRNA-seq, we further validated some of the genes of the monocytic populations in supplementary figure 4a of the immune cell populations and used the scRNA-seq as a validation of the flow cytometry analyses performed in figure 3 for various mice.
Randomization	For the animal studies, tumor volume measurement was obtained for each individual mouse by MRI pre-treatment to constitute treatment groups composed of animals bearing comparably sized tumors.
Blinding	For animal experiments it was difficult to perform blinded treatments, because of the nature of the mouse treatments (anti-IgG2A, anti-GMCSF+PD-L1, anti-VEGF+anti-PD-L1, anti-GM-CSF and anti-GM-CSF+anti-VEGF) applied in experimental groups , However, the measure of tumor volumes was performed blindly as per which group of treatment the animals belong to. Data analysis on collected and digested tissue was done blindly.

Reporting for specific materials, systems and methods

We require information from authors about some types of materials, experimental systems and methods used in many studies. Here, indicate whether each material, system or method listed is relevant to your study. If you are not sure if a list item applies to your research, read the appropriate section before selecting a response.

Materials & experimental systems

n/a	Involved in the study	
☐	☒	Antibodies
☐	☒	Eukaryotic cell lines
☒	☐	Palaeontology and archaeology
☐	☒	Animals and other organisms
☒	☐	Clinical data
☒	☐	Dual use research of concern
☒	☐	Plants

Methods

n/a	Involved in the study	
☒	☐	ChIP-seq
☐	☒	Flow cytometry
☒	☐	MRI-based neuroimaging

Antibodies

Antibodies used	Westernblot antibodies: p-AKT (4060S; CST; 1:1000 dilution), p-c-Jun (3270S; CST; 1:1000 dilution), p-ERK1/2 (4370S;CST;1:500 dilution), T-ERK1/2 (9102S;CST; 1:1000 dilution) , p-S6RP (211S; CST; 1:1000 dilution), p-4EBP1 (9459S;CST; 1:1000 dilution) , Ras-G12D (14429S; CST; 1:500 dilution), p-RSK-1 (9341S;CST; 1:1000 dilution), and vinculin (13901T;CST; 1:1000 dilution): Antibodies used in IHC and flowcytometry in this study are listed in supplementary table 10 and 11 respectively.
Validation	titration assays and fluorescence minus one controls were used to validate the flowcytometry antibodies.<br>positive and negative controls for tissue staining were used to validated the IHC antibodies.<br>positive and negative controls conditions were used to validated the westernblot antibodies.

Eukaryotic cell lines

Policy information about cell lines and Sex and Gender in Research

Cell line source(s)	AML12 cells were provided as a kind gift from Urszula Hibner, HEK-BlueTM cells were obtained from InvivoGen. HEK cells 293T were a kind gift from Karin de Visser lab. HCC cell lines MycOE/Trp53KO, MycOE/PtenKO, NrasG12D/PtenKO, and NrasG12V/PtenKO HCC cell lines were generated a and isolated from end-stage tumor-bearing female mice of each distinct HCC mouse model.
Authentication	MycOE/Trp53KO, MycOE/PtenKO, NrasG12D/PtenKO, and NrasG12V/PtenKO were not authenticated
Mycoplasma contamination	All cell lines were routinely tested negative for mycoplasma contamination

Commonly misidentified lines (See ICLAC register)	no commonly misidentfied cell lines were used

Animals and other research organisms

Policy information about studies involving animals; ARRIVE guidelines recommended for reporting animal research, and Sex and Gender in Research

Laboratory animals	C57BL/6J mice were bred and delivered by Janvier labs. Female mice were used for experimental use at an age of 6-8 weeks. all mice were maintained in pathogen-free facility of the NKI. the mice are housed in individually ventilated cages (IVC) at an ambient temperature of around 20 degrees celsius and humidity around 52% with ad libitum access to laboratory food and water at a 12-h dark/light cycle.
Wild animals	This study did not involve wild animals
Reporting on sex	no sex-or gender based analyses were done. In the interest of ensuring reproducibility of tumor kinetics, penetrance and growth, the animal experiments were conducted using only one gender.
Field-collected samples	This study did not involve samples collected from the field
Ethics oversight	All animal experiments were reviewed and approved by the Animal Ethics Committee of the Netherlands Cancer Institute and performed in accordance with institutional, national and European guidelines for Animal Care and Use. (CCD project id: 28 and 30)

Note that full information on the approval of the study protocol must also be provided in the manuscript.

Plants

Seed stocks	*Report on the source of all seed stocks or other plant material used. If applicable, state the seed stock centre and catalogue number. If plant specimens were collected from the field, describe the collection location, date and sampling procedures.*
Novel plant genotypes	*Describe the methods by which all novel plant genotypes were produced. This includes those generated by transgenic approaches, gene editing, chemical/radiation-based mutagenesis and hybridization. For transgenic lines, describe the transformation method, the number of independent lines analyzed and the generation upon which experiments were performed. For gene-edited lines, describe the editor used, the endogenous sequence targeted for editing, the targeting guide RNA sequence (if applicable) and how the editor was applied.*
Authentication	*Describe any authentication procedures for each seed stock used or novel genotype generated. Describe any experiments used to assess the effect of a mutation and, where applicable, how potential secondary effects (e.g. second site T-DNA insertions, mosiacism, off-target gene editing) were examined.*

Flow Cytometry

Plots

Confirm that:

- [x] The axis labels state the marker and fluorochrome used (e.g. CD4-FITC).
- [x] The axis scales are clearly visible. Include numbers along axes only for bottom left plot of group (a 'group' is an analysis of identical markers).
- [x] All plots are contour plots with outliers or pseudocolor plots.
- [ ] A numerical value for number of cells or percentage (with statistics) is provided.

Methodology

Sample preparation	For ex vivo cell culture experiments cell suspension was harvested and centrifuged at 300 x g for 5 minutes and the cell pellet was collected. For in vivo mouse experiments, macrodissected HCC nodules and control liver samples were dissociated as single-cell suspensions using the Liver Dissociation kit (Miltenyi Biotec) and the gentleMACS Octo Dissociator following the manufacturer’s instructions. The cell pellet of ex vivo and in vivo samples were incubated with anti-CD16/CD32 antibody (BD Bioscience) and stained with the antibodies against surface markers following standard procedures. Samples were fixed with the eBioscience fixation and permeabilization kit (Invitrogen), and stained for intracellular markers (see Supplementary Table 10)
Instrument	Samples were acquired using a BD LSRFortessas TM (BD BioSciences) or a Cytek Aurora (Cytek) and cells were sorted using a FACSAria Fusion (BD BioSciences).
Software	Data acquisition was performed using DIVA software (BD Biosciences) or Spectroflo (Cytek). Data analysis including quantification and data visualisation were performed using FlowJo Software version 10.8.1 (BD BioSciences) and Graphpad Prism 9.0.0 (Graphpad software).

Cell population abundance	For the RT-qPCR analysis 30,000 MDMs and 30,000 Ly6Chigh monocytes were sorted per sample using the following gating stratgy (MDMs) CD45+CD11b+Ly6C–Ly6G–F4/80int and (Ly6Chigh) CD45+CD11b+Ly6G–Ly6Chigh. for CD45+ sequencing atleast 30,000 CD45+ cells were sorted per sample. for scRNA seq atleast 30,000 CD45- and CD45+ cells were sorted per sample.
Gating strategy	To determine the populations of myeloid and lymphoid cells, we first used SSC, FSC and Zombie-NIR (live/dead dye) gating to select for single cell and live cell events. Immune cell populations were then identified by their high expression levels of CD45.<br><br>The lymphoid panel consist of CD45+ cells that were CD11b-.<br>CD8 T cells were gated according to the following marker expression: NK1.1-CD19-CD8+ from the parental CD45+CD3+ population of lymphoid cells<br>CD4 T cells were gated according to the following marker expression: NK1.1-CD19-CD4+ from the parental CD45+CD3+ population of lymphoid cells<br><br>The myeloid panel consist of double positive CD45+ and CD11bhigh cells.<br>Dendritic cells (DCs) were gated according to the following marker expression: CD11c+MHCII+ from the parental CD45 +F4/80- population of myeloid cells<br>Ly6Chigh monocytes were gated according to the following marker expression: Ly6G-Ly6Chigh from the parental CD45+ and CD11bhigh population of myeloid cells<br>Ly6Clow monocytes were gated according to the following marker expression: Ly6G-Ly6Clow from the parental CD45+ and CD11bhigh population (longitudinal blood analysis)<br>Neutrophils were gated according to the following marker expression Ly6Cinter Ly6G+ from the parental CD45+ and CD11b+ population of myeloid cells<br>Tumor associated macrophages (TAMs) were gated according to the following marker expression: Ly6C-Ly6G-F4/80int/high from the CD45+ and CD11b+ population of myeloid cells<br>Monocyte-derived macrophages (MDMs) were gated according to the following marker expression: Ly6ClowLy6G-F4/80int from the CD45+ and CD11bhigh population of myeloid cells.<br>Kupffer cells (KCs) were gated according to the following marker expression: Ly6ClowLy6G-F4/80high from the CD45+ and CD11bint population of myeloid cells.<br>Ly6Clow cells were gated according to the following marker expression: Ly6ClowLy6G-F4/80low from the CD45+ and CD11bhigh population of myeloid cells.<br><br>The myeloid and lymphoid panels were optimized with titrated antibodies and fluorescence minus one (FMO) controls were used to set up positive and negative populations.

☒ Tick this box to confirm that a figure exemplifying the gating strategy is provided in the Supplementary Information.
