# Supplementary material for: Cancer cell genetics shaping of the tumor microenvironment reveals myeloid cell-centric exploitable vulnerabilities in hepatocellular carcinoma
Source: Nat Commun. 2024 Mar 22;15:2581. doi: 10.1038/s41467-024-46835-2 (PMC10959959; doi:10.1038/s41467-024-46835-2)
Supplement: Supplementary file 1 — Supplementary Information [file 41467_2024_46835_MOESM1_ESM.pdf]

Supplementary figure 1

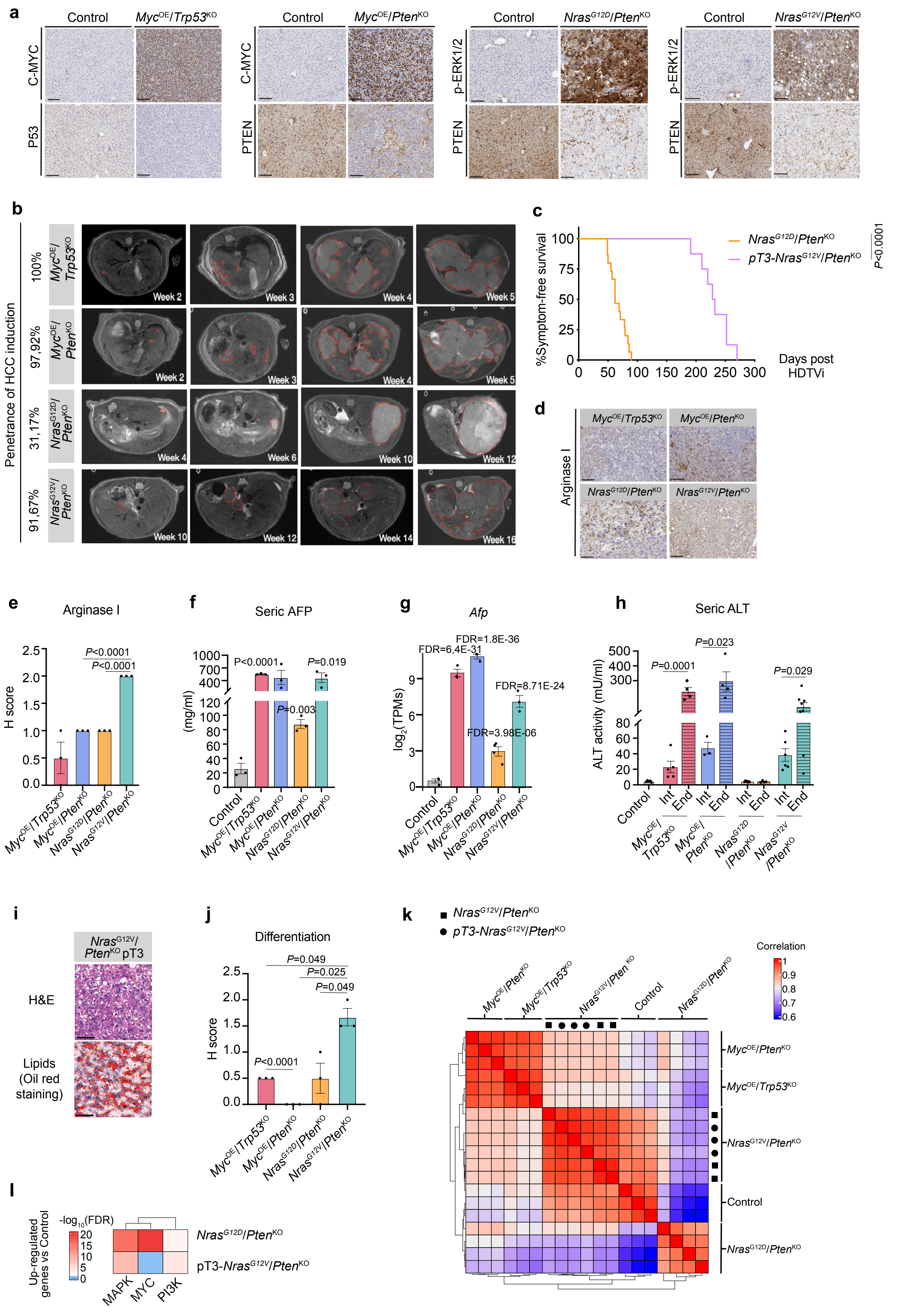

Supplementary Figure 1: Genetically-distinct HCCs display different features of malignant transformation.

a. Representative IHC images of C-MYC, P53, PTEN and phospho-ERK1/2 staining performed on liver sections from control and HCC-bearing mice (Scale bars=100 µm; representative of *n*=4 mice).

b. MRI scans illustrating tumor penetrance, latency and growth in HCC models. Red lines indicate the regions of interest to calculate the tumor volumes presented in Fig. 1c.

c. Kaplan–Meier curves of *Nras*^{G12D}/*Pten*^{KO} (*n*=15; median survival=62 days also shown in Fig. 1b), and *pT3-Nras*^{G12V}/*Pten*^{KO} (*n*=8; median survival=230 days) HCC-bearing mice.

d. Representative IHC images of Arginase-1 staining performed on liver sections from end-stage HCC-bearing mice (Scale bars=100 µm; representative of *n*=3 mice).

e. Histopathology scoring of Arginase-1 in end-stage HCC (presented in (d); *n*=3 for all HCC models).

f. AFP quantification performed in the sera of control and HCC-bearing mice at intermediate stage (*n*=3 for all models).

g. *Afp* expression in control (*n*=3) and end-stage HCCs (*Myc*^{OE}/*Trp53*^{KO} *n*=3, *Myc*^{OE}/*Pten*^{KO} *n*=3, *Nras*^{G12D}/*Pten*^{KO} *n*=4, *Nras*^{G12V}/*Pten*^{KO} *n*=3) from the bulk RNA-seq.

h. Seric ALT activity in control (*n*=5) and HCC-bearing mice (*Myc*^{OE}/*Trp53*^{KO} intermediate [Int] *n*=5 and end [End]-stage *n*=4, *Myc*^{OE}/*Pten*^{KO} Int *n*=3 and End *n*=4, *Nras*^{G12D}/*Pten*^{KO} Int *n*=7 and End *n*=4, *Nras*^{G12V}/*Pten*^{KO} Int *n*=6 and End *n*=8).

i. Representative images of H&E and Oil Red staining performed on sectioned livers from end-stage *pT3-Nras*^{G12V}/*Pten*^{KO} tumor-bearing mice (Scale bars=100 µm; representative of *n*=5 animals).

j. Differentiation status of end-stage HCCs according to the 2019 WHO classification histopathology grading guidelines[1] (*n*=3 for all models).

k. Unsupervised hierarchical clustering of the Pearson correlation calculated from the normalized Transcripts per Million (TPMs) of control (*n*=3) and HCCs (*n*=3 for *Myc*^{OE}/*Trp53*^{KO} and *Myc*^{OE}/*Pten*^{KO} , *n*=6 for *Nras*^{G12V}/*Pten*^{KO}, *n*=4 for *Nras*^{G12D}/*Pten*^{KO}).

l. Unsupervised hierarchical clustering of the geneset enrichment for the indicated signaling pathways in *Nras*^{G12D}/*Pten*^{KO} (shown in Fig. 1f, *n*=3) and *pT3-Nras*^{G12V}/*Pten*^{KO} (*n*=3) HCCs

compared to control. The color scale represents the significance in $-\log_{10}$(FDR). FDR False Discovery Rate.

Graphs show the mean±SEM (e-h, j). Statistical significance: log-rank (mantel-cox) test (c), one-way ANOVA with Tukey's multiple comparison test (e, j), differential gene expression (g), unpaired two-sided Student's *t*-test (f, h), two-sided hypergeometric test with multiple testing correction using Benjamini-Hochberg (l). Source data are provided as a Source Data file.

Supplementary figure 2

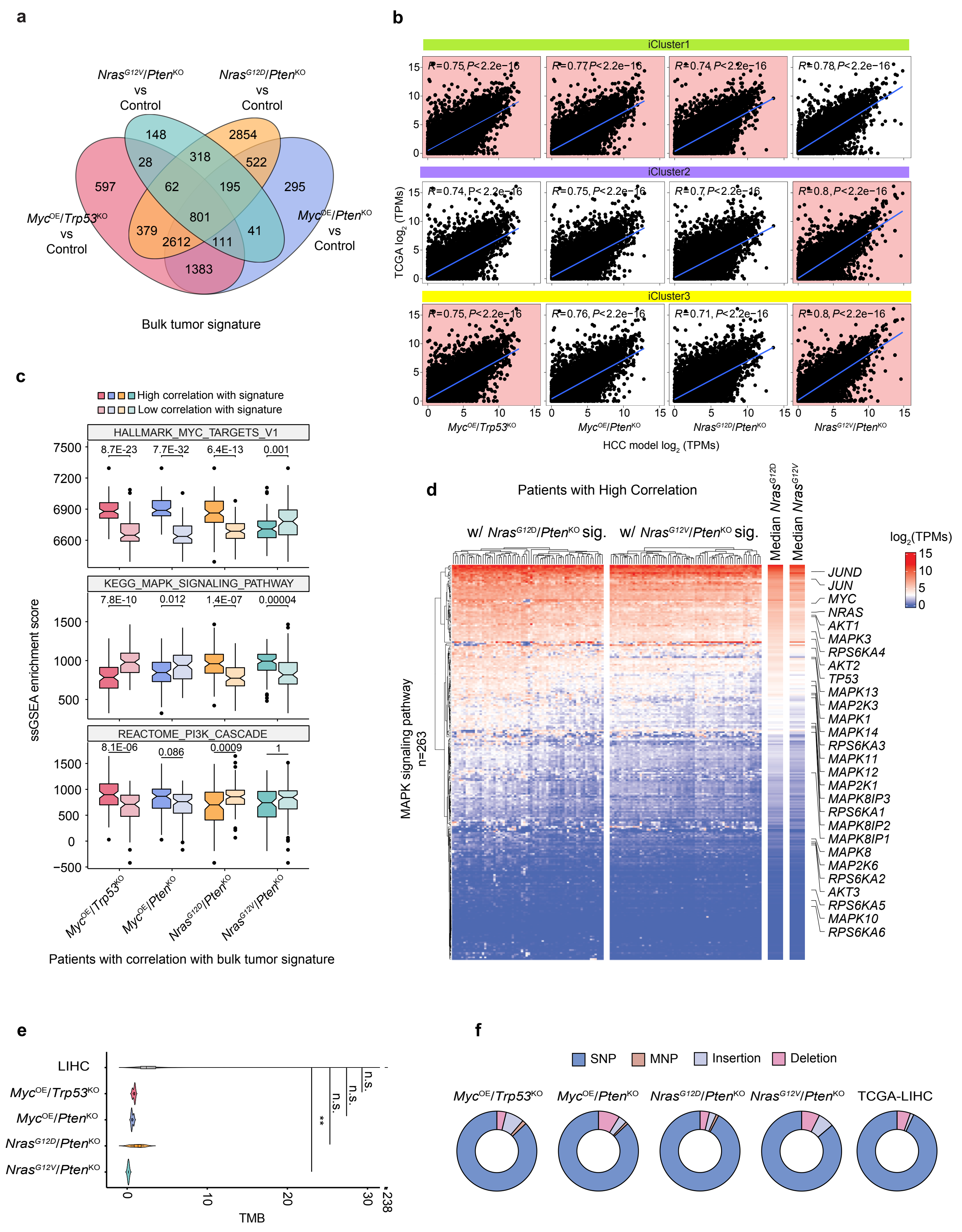

Supplementary Figure 2: HCC patients with matching profiles to genetically-distinct murine models display distinct molecular and prognostic features.

a. Venn diagram of the differentially expressed genes (DEG) between genetically-distinct HCCs (*Myc*^{OE}/*Trp53*^{KO} *n*=3, *Myc*^{OE}/*Pten*^{KO} *n*=3, *Nras*^{G12D}/*Pten*^{KO} *n*=4, *Nras*^{G12V}/*Pten*^{KO} *n*=3) and control livers (*n*=3) as determined in RNA-seq analyses (see Supplementary Data 1).

b. Scatterplots depicting the correlation in global gene expression between TCGA:LIHC patients (*n*=423) and each of the genetically-distinct HCC tumors (*Myc*^{OE}/*Trp53*^{KO} *n*=3, *Myc*^{OE}/*Pten*^{KO} *n*=3, *Nras*^{G12D}/*Pten*^{KO} *n*=4, *Nras*^{G12V}/*Pten*^{KO} *n*=3). TCG:LIHC patients were divided according to their TCGA classification[2].

c. Boxplots depicting the enrichment of the MYC, MAPK and PI3K/mTOR signaling pathways in patients segregated according to their high/low correlation with each of the genetically-distinct HCC bulk tumor transcriptional signatures from Fig. 1e (sample size *n*=93 for each set of patients).

d. Heatmap showing the expression of MAPK signaling pathway genes in TCGA:LIHC patients[2] (*n*= 263) presenting high enrichment of this pathway and that also have high correlation with *Nras*^{G12D}/*Pten*^{KO} (left) or *Nras*^{G12V}/*Pten*^{KO} (right) signatures.

e. Violin plots depicting the tumor mutational burden (TMB) of genetically-distinct HCC compared to human TCGA:LIHC[2] (*n*=723), as determined by whole exome sequencing analyses of bulk tumors (*n*=3 for *Myc*^{OE}/*Trp53*^{KO}, *Myc*^{OE}/*Pten*^{KO}, and *Nras*^{G12D}/*Pten*^{KO}, *n*=4 for (*pT3-*) *Nras*^{G12V}/*Pten*^{KO}) (see Supplementary Data 3).

f. Donut charts of the variant type distribution of mutations of genetically-distinct HCC (*n*=3 per genotype) compared to TCGA-LIHC[2] (*n*=371). SNP: single nucleotide polymorphism. MNP: multiple nucleotide polymorphism.

Statistical significance was determined by a two-sided Student's t-test for association between paired samples, using Pearson's product moment correlation coefficient (b), a paired two-sided Student's t-test with Bonferroni multiple testing correction (c) and an unpaired two-sided Wilcoxon test (e). $^{**}p < 0.01$, n.s. non-significant ($p \geq 0.01$). Source data are provided as a Source Data file.

Supplementary figure 3

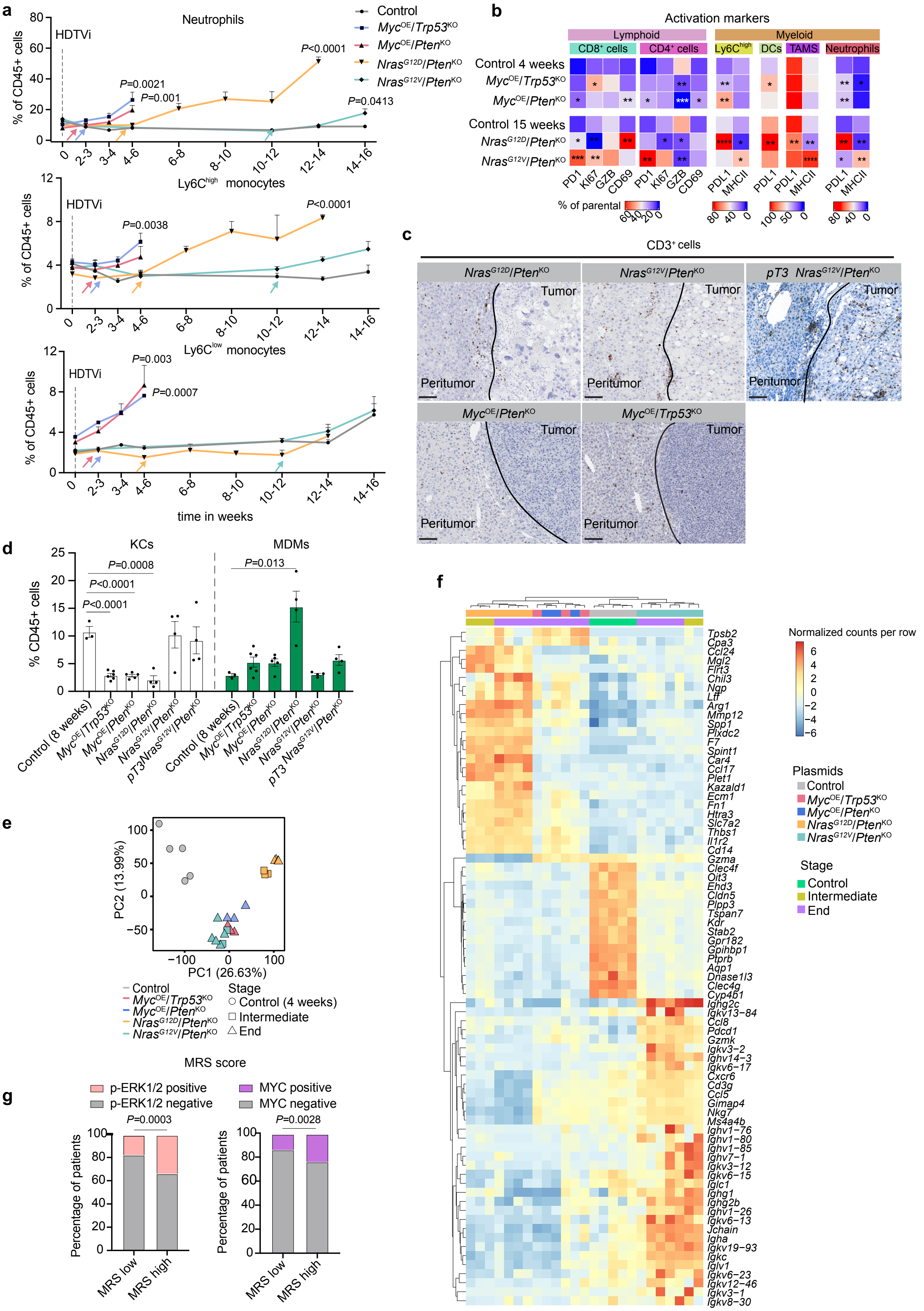

h

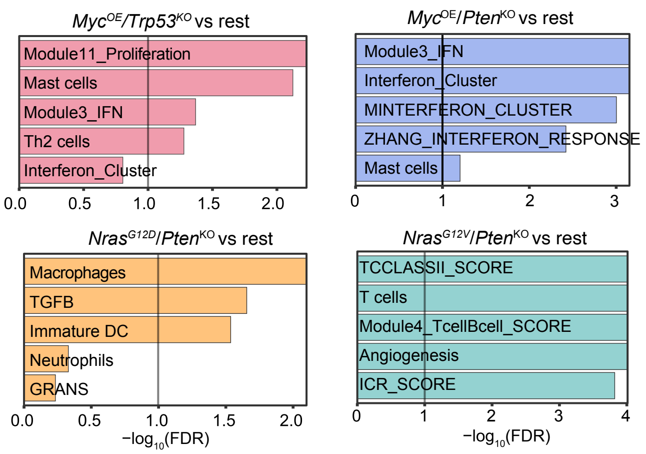

MycOE/Trp53KO vs rest
Module11_Proliferation
Mast cells
Module3_IFN
Th2 cells
Interferon_Cluster
0.0 0.5 1.0 1.5 2.0
MycOE/PtenKO vs rest
Module3_IFN
Interferon_Cluster
MINTERFERON_CLUSTER
ZHANG_INTERFERON_RESPONSE
Mast cells
0 1 2 3
NrasG12D/PtenKO vs rest
Macrophages
TGFB
Immature DC
Neutrophils
GRANS
0.0 0.5 1.0 1.5 2.0
−log10(FDR)
NrasG12V/PtenKO vs rest
TCCLASSII_SCORE
T cells
Module4_TcellBcell_SCORE
Angiogenesis
ICR_SCORE
0 1 2 3 4
−log10(FDR)

i

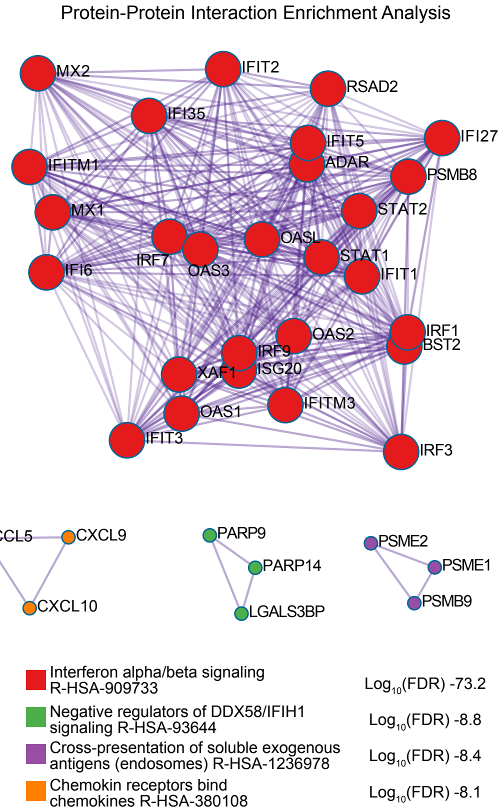

Protein-Protein Interaction Enrichment Analysis
MX2
IFIT2
RSAD2
IFI35
IFIT5
IFI27
IFITM1
ADAR
PSMB8
MX1
STAT2
OASL
IRF7
OAS3
STAT1
IFI6
IFIT1
OAS2
IRF1
BST2
IRF9
XAF1
ISG20
OAS1
IFITM3
IFIT3
IRF3
CCL5
CXCL9
CXCL10
PARP9
PARP14
LGALS3BP
PSME2
PSME1
PSMB9
Interferon alpha/beta signaling R-HSA-909733
Log10(FDR) -73.2
Negative regulators of DDX58/IFIH1 signaling R-HSA-93644
Log10(FDR) -8.8
Cross-presentation of soluble exogenous antigens (endosomes) R-HSA-1236978
Log10(FDR) -8.4
Chemokin receptors bind chemokines R-HSA-380108
Log10(FDR) -8.1

j

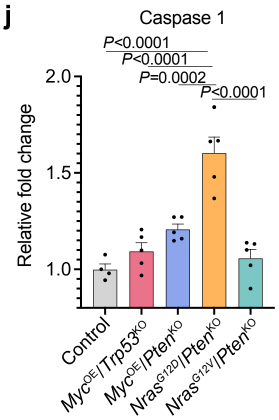

Caspase 1
P<0.0001
P<0.0001
P=0.0002
P<0.0001
Relative fold change
2.0
1.5
1.0
Control
MycOE/Trp53KO
MycOE/PtenKO
NrasG12D/PtenKO
NrasG12V/PtenKO

Supplementary Figure 3: Cancer cell-intrinsic signaling pathways dynamically change HCC immune landscape.

a. Longitudinal quantification of circulating neutrophils, Ly6C^{high} monocytes and Ly6C^{low} monocytes at the indicated time points post-HDTVi (Control *n*=10, *Myc*^{OE}/*Trp53*^{KO} *n*=20, *Myc*^{OE}/*Pten*^{KO} *n*=18, *Nras*^{G12D}/*Pten*^{KO} *n*=30, and *Nras*^{G12V}/*Pten*^{KO} *n*=16). Arrows indicate the time points when tumors were detected. Statistical significance tested against aged-matched control livers.

b. Median percentage of immune cell populations in end-stage HCC expressing the indicated phenotypic markers. Statistical significance tested against aged-matched control livers *p < 0.05; **p < 0.01; ***p < 0.001; ****p < 0.0001 (see source data for number of replicates).

c. Representative CD3 IHC staining performed in liver sections from HCC-bearing mice. Black lines indicate the tumor borders (Scale bars=100 µm; representative of *n*=3 mice for each model).

d. Percentages of KCs and MDMs (Control *n*=3, *Myc*^{OE}/*Trp53*^{KO} *n*=6, *Myc*^{OE}/*Pten*^{KO} *n*=5, *Nras*^{G12D}/*Pten*^{KO} *n*=4, *Nras*^{G12V}/*Pten*^{KO} *n*=4, *pT3-Nras*^{G12V}/*Pten*^{KO} *n*=4).

e. PCA plot depicting control livers and HCCs CD45^{+} immune cell transcriptome following RNA-seq analyses (Control *n*=5, *Myc*^{OE}/*Trp53*^{KO} *n*=3; *Myc*^{OE}/*Pten*^{KO} *n*=3; *Nras*^{G12D}/*Pten*^{KO} intermediate *n*=3, end-stage *n*=4; *Nras*^{G12V}/*Pten*^{KO} intermediate *n*=2, end-stage *n*=5).

f. Unsupervised hierarchical clustering of the top 60 differently expressed genes in the CD45^{+} immune cell transcriptome of control and HCC-bearing mice (presented in (e)).

g. Myeloid responsive score (MRS) in HCC patients[3] segregated according to p-ERK1/2 (top) (negative *n*=369 and positive *n*=99) and MYC (bottom) (negative *n*=389 and positive *n*=78) expressing cancer cells.

h. Enrichment for specific immune genesets in end-stage HCCs CD45^{+} cells compared to the rest of the HCC models (vs rest) (*Myc*^{OE}/*Trp53*^{KO} *n*=3; *Myc*^{OE}/*Pten*^{KO} *n*=3; *Nras*^{G12D}/*Pten*^{KO} *n*=4; *Nras*^{G12V}/*Pten*^{KO} *n*=5). Vertical line at $-\log_{10}$(FDR)=1 indicates the significance threshold.

i. Protein-protein interaction enrichment analyses from the interferon response genes identified in the *Myc*^{OE}-HCCs (presented in (h)). Node size represents the $-\log_{10}$ (FDR). FDR False Discovery Rate.

j. Caspase 1 activity level in control (*n*=4) and end-stage HCCs (*n*=5 for each model). Results are shown as relative fold change compared to control.

Graphs show mean+SEM (a, j) and ±SEM (d). Statistical significance: two-sided unpaired Student's *t*-test analysis in (a-b, d), χ^2 test in (g), two-sided weighted Kolmogorov-Smirnov statistic test (h), two-sided hypergeometric test with Benjamini-Hochberg multiple testing correction (i), one-way ANOVA Tukey's multiple comparisons test (j). See Supplementary Fig. 9k-j for gating strategy (a,b,d). Source data are provided as a Source Data file.

Supplementary figure 4

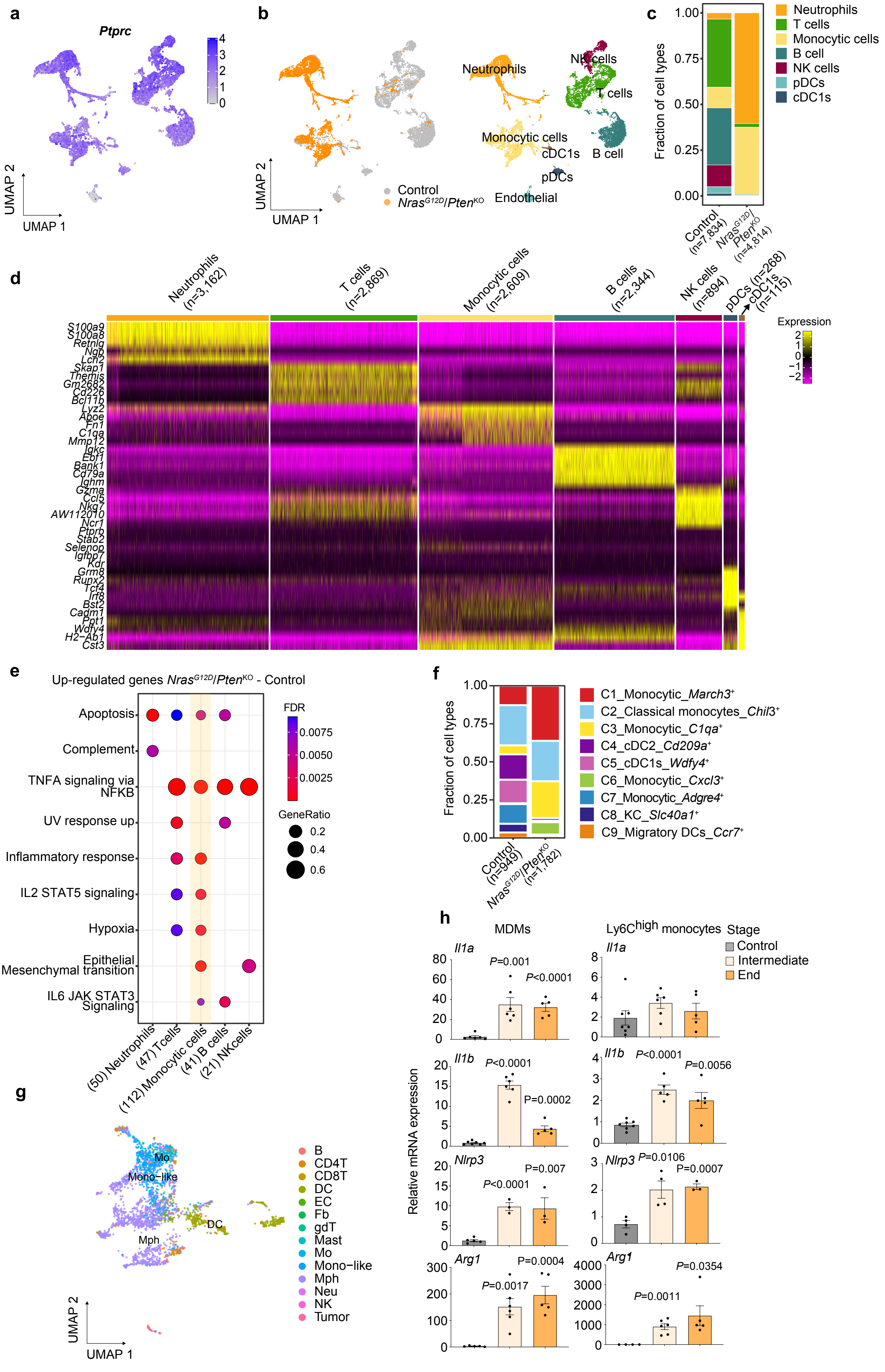

Supplementary Figure 4: In-depth analysis of *Nras*^{G12D}/*Pten*^{KO} tumor immune landscape.

a. *Ptprc* (CD45) gene expression levels as identified in the scRNA-seq dataset of control liver (*n*=1) and *Nras*^{G12D}/*Pten*^{KO} HCC (*n*=1) shown in Fig. 4a.

b. UMAP representation as shown in Fig. 4a including the 'Endothelial cells' cluster.

c. Barplot depicting the proportions of the indicated immune cell subsets as identified in the scRNA-seq dataset of control liver and *Nras*^{G12D}/*Pten*^{KO} HCC.

d. Heatmap displaying the expression levels of the top 10 most differentially expressed genes per immune cell subset and their expression across the other immune clusters from the scRNA-seq dataset in Fig. 4a.

e. Bubble plot showing the gene set enrichment of up-regulated genes in *Nras*^{G12D}/*Pten*^{KO} HCC compared to control liver in the indicated immune cell subsets. Circle size represents the ratio of overlapping genes between the gene set and the DEGs. Significance threshold of FDR<0.05 (see Supplementary Data 8). FDR False Discovery Rate.

f. Barplots depicting the proportions of the indicated myeloid cell subsets as identified in the 'Monocytic cell' cluster in control liver and *Nras*^{G12D}/*Pten*^{KO} HCC scRNA-seq dataset (Fig. 4d).

g. UMAP of the subclusters from the 'Monocytic cell' population in control liver and *Nras*^{G12D}/*Pten*^{KO} HCC scRNA-seq dataset (Fig. 4d) displaying annotations from human HCC scRNA-seq dataset[4] subclusters after integration of both datasets.

h. Barplots depicting the relative mRNA expression levels of indicated genes in FACS-isolated MDMs from control liver and *Nras*^{G12D}/*Pten*^{KO} HCC (*Il1a*; control *n*=6, *intermediate n*=6, end *n*=5, *Il1b*; control *n*=7, *intermediate n*=6, end *n*=5, *Nlrp3*; control *n*=5, *intermediate n*=3, end *n*=3, *Arg1*; control *n*=5 *intermediate n*=6, end *n*=5) and Ly6C^{high} monocytes (*Il1a*; control *n*=7, *intermediate n*=6, end *n*=5, *Il1b*; control *n*=7, *intermediate n*=5, end *n*=5 *Nlrp3*; control *n*=4, *intermediate n*=4, end *n*=3, *Arg1*; control *n*=4, *intermediate n*=6, end *n*=5) at intermediate and end stages. *Ubc* was used as a housekeeping gene in all analyses. All samples are relative to one control liver sample.

Graphs show mean±SEM (h). Statistical significance was determined by two-sided unpaired Student's *t*-test (h). See Supplementary Figure 9j for gating strategy of FACS-isolation (h). Source data are provided as a Source Data file.

Supplementary figure 5

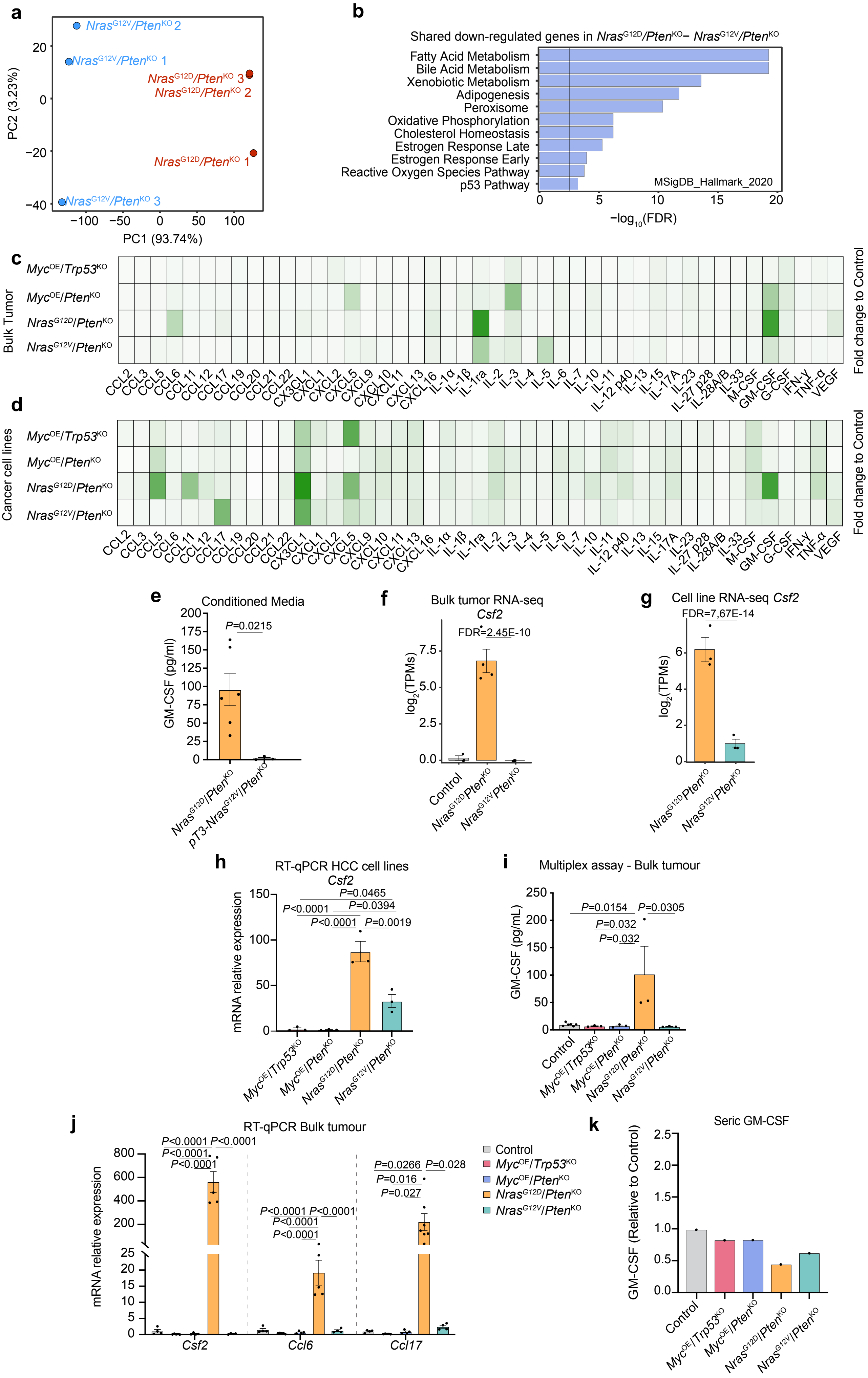

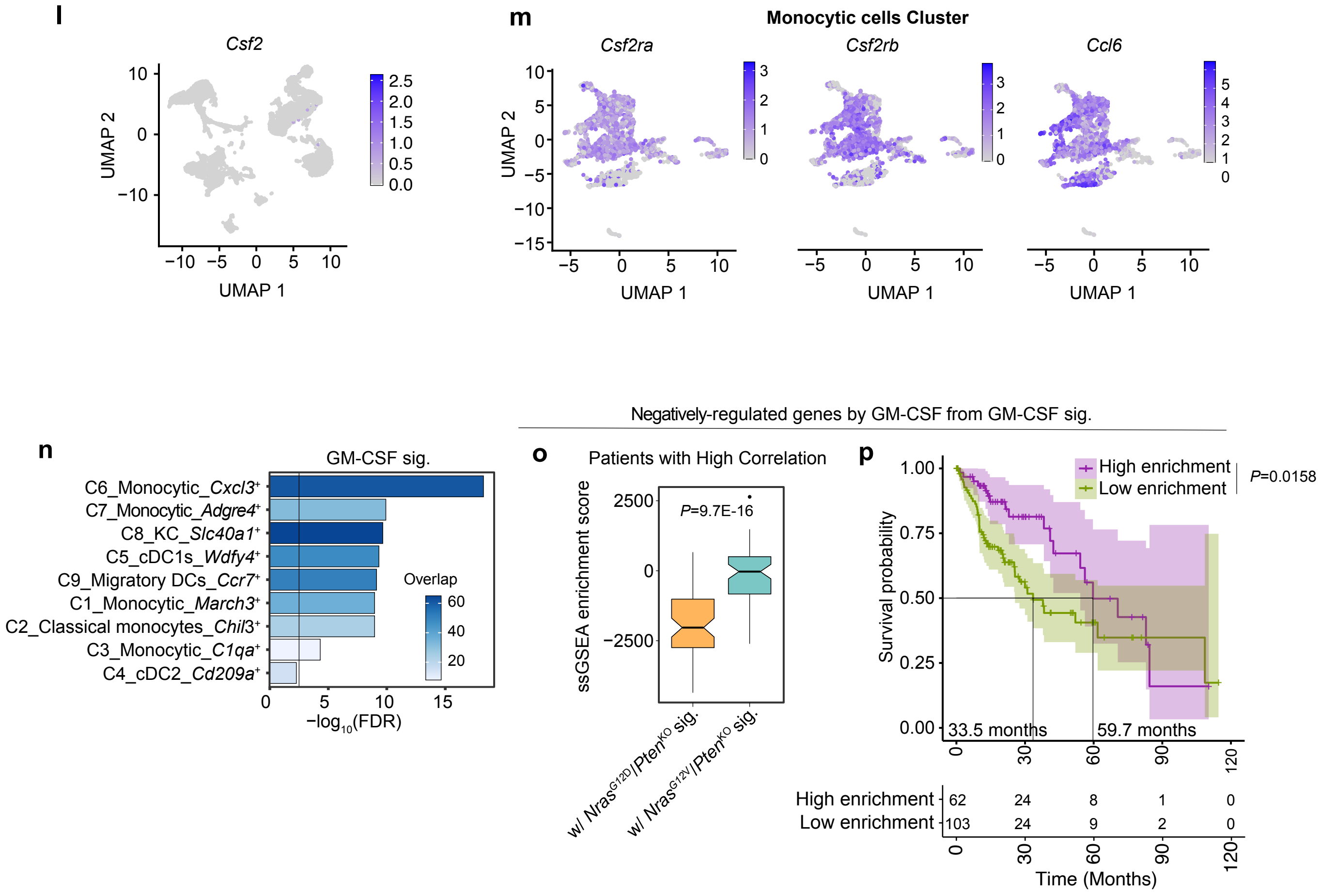
l
Csf2
UMAP 2
UMAP 1
m
Monocytic cells Cluster
Csf2ra
Csf2rb
Ccl6
n
GM-CSF sig.
C6_Monocytic_Cxcl3+
C7_Monocytic_Adgre4+
C8_KC_Slc40a1+
C5_cDC1s_Wdfy4+
C9_Migratory DCs_Ccr7+
C1_Monocytic_March3+
C2_Classical monocytes_Chil3+
C3_Monocytic_C1qa+
C4_cDC2_Cd209a+
Overlap
−log10(FDR)
Negatively-regulated genes by GM-CSF from GM-CSF sig.
o
Patients with High Correlation
ssGSEA enrichment score
P=9.7E-16
w/ NrasG12D/PtenKO sig.
w/ NrasG12V/PtenKO sig.
p
High enrichment
Low enrichment
P=0.0158
Survival probability
33.5 months
59.7 months
High enrichment 62 24 8 1 0
Low enrichment 103 24 9 2 0
Time (Months)

Supplementary Figure 5: GM-CSF pathway underlies reciprocal interaction between *Nras*^{G12D}/*Pten*^{KO} cancer cells and TME.

a. *Nras*^{G12D}/*Pten*^{KO} and *Nras*^{G12V}/*Pten*^{KO} cancer cell transcriptomes (*n*=3 per cell line).

b. Signaling pathways enriched in down-regulated genes shown in Fig. 5a (blue colored dots).

c-d. Proteomic profile analyses of bulk tumors (c) or conditioned media from cancer cells (d) (*n*=1), relative to control livers or AML12 cells, respectively. Values are shown as pixel density fold changes compared to controls.

e. GM-CSF level in conditioned media of *Nras*^{G12D}/*Pten*^{KO} (*n*=6, from Fig. 5d) and *pT3-Nras*^{G12V}/*Pten*^{KO} cells (*n*=3).

f-g. *Csf2* expression from RNA-seq of control livers (*n*=3), bulk *Nras*^{G12D}/*Pten*^{KO} (*n*=4) and *Nras*^{G12V}/*Pten*^{KO} (*n*=3) tumors (f), or *Nras*^{G12D}/*Pten*^{KO} (*n*=3) and *Nras*^{G12V}/*Pten*^{KO} (*n*=3) cancer cells (g).

h. *Csf2* expression by RT-qPCR in *Myc*^{OE}/*Trp53*^{KO}, *Myc*^{OE}/*Pten*^{KO}, *Nras*^{G12D}/*Pten*^{KO} and *Nras*^{G12V}/*Pten*^{KO} cancer cells (*n*=3 each). One *Myc*^{OE}/*Trp53*^{KO} sample is set as baseline.

i. GM-CSF levels by Luminex assay in control livers (*n*=6), and end-stage genetically-distinct HCCs (*n*=3 for each tumor model).

j. *Csf2*, *Ccl6* and *Ccl17* expression by RT-qPCR in control (*n*=4), *Myc*^{OE}/*Trp53*^{KO} (*n*=5), *Myc*^{OE}/*Pten*^{KO} (*n*=4), *Nras*^{G12D}/*Pten*^{KO} (for *Csf2* and *Ccl6* *n*=5 and for *Ccl17* *n*=7) and *Nras*^{G12V}/*Pten*^{KO}(*n*=4) end-stage bulk tumors. One control liver is set as baseline for all genes.

k. Relative pixel density of GM-CSF levels by proteomic profile analyses of control mouse and genetically-distinct HCC-bearing mice sera (*n*=1 each). Control sample is set as baseline.

l-m. *Csf2* expression in all populations (l) and *Csf2ra, Csf2rb* and *Ccl6* expression in 'Monocytic cell' clusters (m) identified by scRNA-seq.

n. GM-CSF signature[5] enrichment in 'Monocytic cells' subclusters identified by scRNA-seq

o. GM-CSF enrichment of negatively-regulated genes (*n*=709) from the GM-CSF signature[5] in TCGA:LIHC patients[2] segregated as high correlation with *Nras*^{G12D}/*Pten*^{KO} or *Nras*^{G12V}/*Pten*^{KO} transcriptional signatures (sample size as Fig. 5i).

p. Kaplan-Meier curves of TCGA-LIHC[2] patients segregated as high/low enrichment of GM-CSF negatively regulated genes (*n*=709) from GM-CSF signature[5] (High *n*=62, Low *n*=103). Table shows live patient numbers at indicated time points exhibiting high or low enrichment of GM-CSF negatively regulated genes.

Graphs show mean+SEM (i) and ±SEM (e-h, j). Statistical significance: two-sided hypergeometric test with multiple testing correction using Benjamini-Hochberg (b, n), differential expression analyses (f, g) one-way ANOVA Tukey's multiple comparisons test (h-j), unpaired two-sided Student's t-test (e, o) and log-rank test (p). Vertical lines at $-\log_{10}(FDR)=2.5$ indicate the threshold for (b and n). FDR False Discovery Rate. The shading represents 95% confidence interval (p). Source data are provided as a Source Data file.

Supplementary figure 6

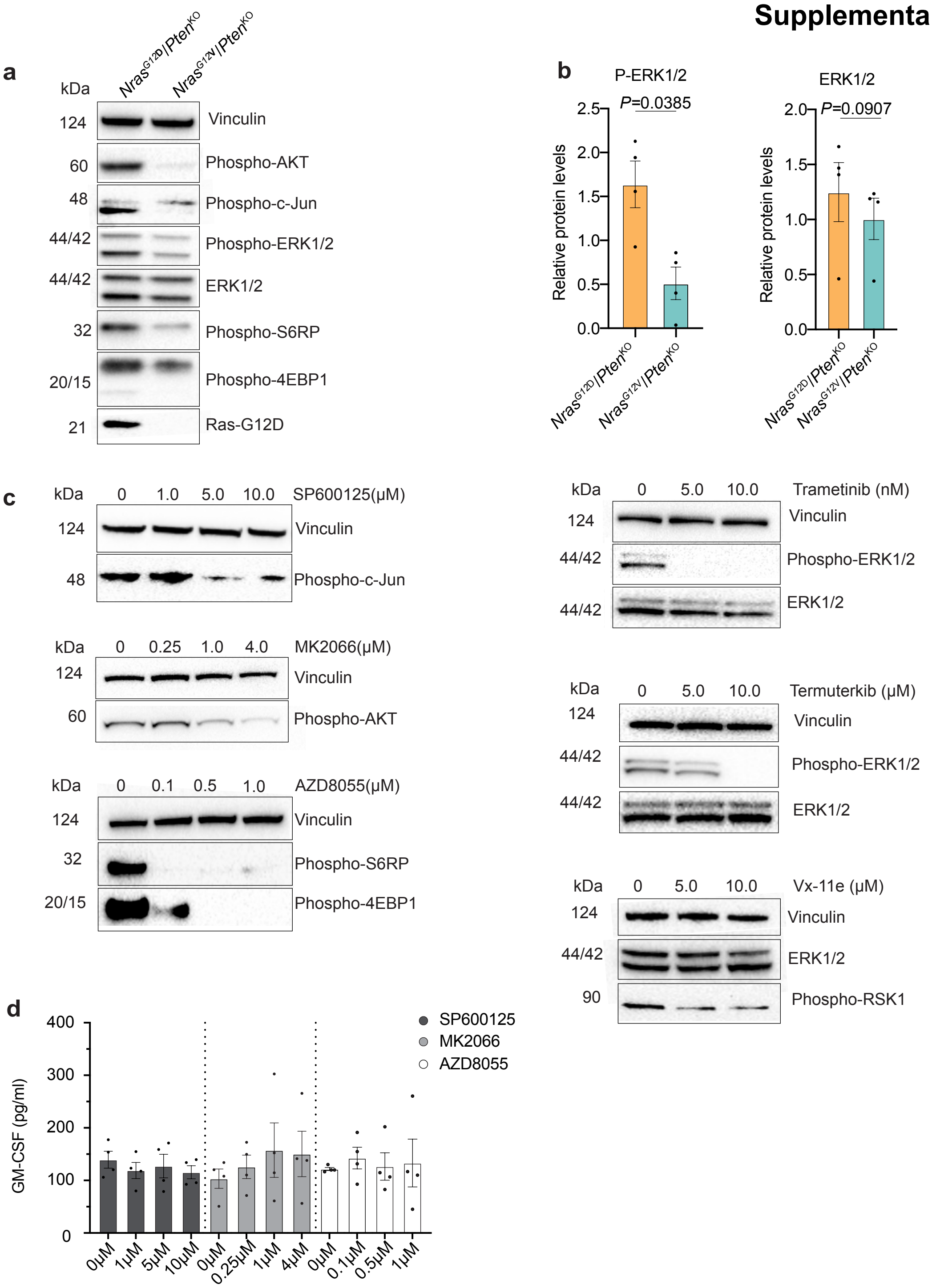

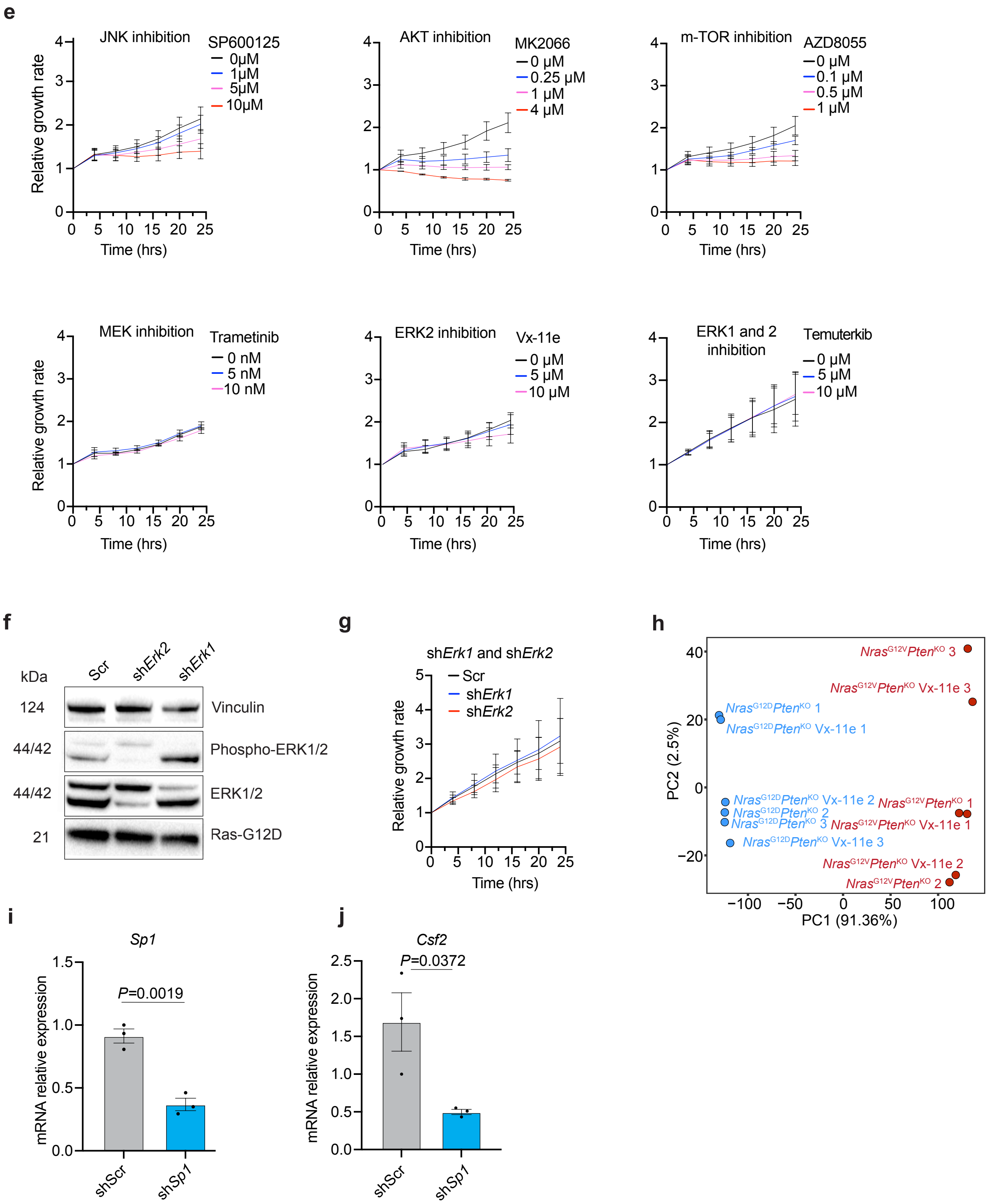
e
JNK inhibition
SP600125
0μM
1μM
5μM
10μM
Relative growth rate
Time (hrs)
AKT inhibition
MK2066
0 μM
0.25 μM
1 μM
4 μM
m-TOR inhibition
AZD8055
0 μM
0.1 μM
0.5 μM
1 μM
MEK inhibition
Trametinib
0 nM
5 nM
10 nM
ERK2 inhibition
Vx-11e
0 μM
5 μM
10 μM
ERK1 and 2 inhibition
Temuterkib
0 μM
5 μM
10 μM
f
kDa
Scr
shErk2
shErk1
124
Vinculin
44/42
Phospho-ERK1/2
44/42
ERK1/2
21
Ras-G12D
g
shErk1 and shErk2
Scr
shErk1
shErk2
Relative growth rate
Time (hrs)
h
Nras G12V Pten KO 3
Nras G12V Pten KO Vx-11e 3
Nras G12D Pten KO 1
Nras G12D Pten KO Vx-11e 1
Nras G12D Pten KO Vx-11e 2
Nras G12V Pten KO 1
Nras G12D Pten KO 2
Nras G12D Pten KO 3
Nras G12V Pten KO Vx-11e 1
Nras G12D Pten KO Vx-11e 3
Nras G12V Pten KO Vx-11e 2
Nras G12V Pten KO 2
PC2 (2.5%)
PC1 (91.36%)
i
Sp1
P=0.0019
mRNA relative expression
shScr
shSp1
j
Csf2
P=0.0372
mRNA relative expression
shScr
shSp1

Supplementary Figure 6: *Nras*^{G12D}-induced MAPK-ERK1/2 pathway specifically modulates GM-CSF levels via SP1.

a. Representative western blots assessing the expression of the indicated proteins in *Nras*^{G12D}/*Pten*^{KO} and *Nras*^{G12V}/*Pten*^{KO} HCC cell lines (representative of *n*=4 independent experiments).

b. Barplots depicting the expression levels of phosphorylated and total ERK1/ERK2 proteins relative to vinculin (presented in a, *n*=4 per cell line).

c. Representative western blots assessing the expression of the indicated proteins in *Nras*^{G12D}/*Pten*^{KO} HCC cell lines after 24h of treatment with SP600125 (JNK inhibitor *n*=4), MK2066 (AKT inhibitor *n*=4), AZD8055 (mTOR inhibitor *n*=4), Trametinib (MEK1/2 inhibitor *n*=4), Temuterkib (ERK1/2 inhibitor *n*=3) and VX-11e (ERK2 inhibitor *n*=4) at the indicated concentrations.

d. Barplots depicting the GM-CSF protein levels quantified in *Nras*^{G12D}/*Pten*^{KO} HCC cell line conditioned media after 24h of treatment with SP600125 (JNK inhibitor; *n*=4), MK2066 (AKT inhibitor; *n*=4) and AZD8055 (m-TOR inhibitor; *n*=4) at the indicated drug concentrations.

e. Growth curves showing the proliferation of *Nras*^{G12D}/*Pten*^{KO} cancer cells treated with Trametinib, Temuterkib, Vx-11e, SP600125, MK2066 and AZD8055 at the indicated concentrations (*n*=3 independent experiments). Growth rate is relative to the 0h timepoint.

f. Representative western blots assessing the expression of the indicated proteins in *Nras*^{G12D}/*Pten*^{KO} HCC cell line expressing either a non-targeting control shRNA (sh*Scr*), an shRNA directed against *Erk1* (sh*Erk1*), or *Erk2* (sh*Erk2*) (representative of *n*=3 independent experiments).

g. Growth curves showing the proliferation of sh*Scr*, sh*Erk1* and s*hErk2 Nras*^{G12D}/*Pten*^{KO} cancer cell lines (*n*=3 independent experiments). Growth rate is relative to the 0h timepoint.

h. PCA plot depicting the transcriptome of genetically-distinct HCC cell lines (*Nras*^{G12D}/*Pten*^{KO}, *Nras*^{G12V}/*Pten*^{KO}) treated with Vx-11e and control (*n*=3 per cell line), following RNA-seq analyses.

i-j. Gene expression analysis of *Sp1* (i) and *Csf2* (j) performed in sh*Scr* (*n*=3) or sh*Sp1* (*n*=3) *Nras*^{G12D}/*Pten*^{KO} cells. *Ubc* was used as a house keeping gene. All samples are relative to one sh*Scr* sample.

Graph shows mean±SEM (b, d, e, g, i-j). Statistical significance was determined by paired (b) and unpaired (i-j) two-sided Student's t-test. Source data are provided as a Source Data file.

Supplementary figure 7

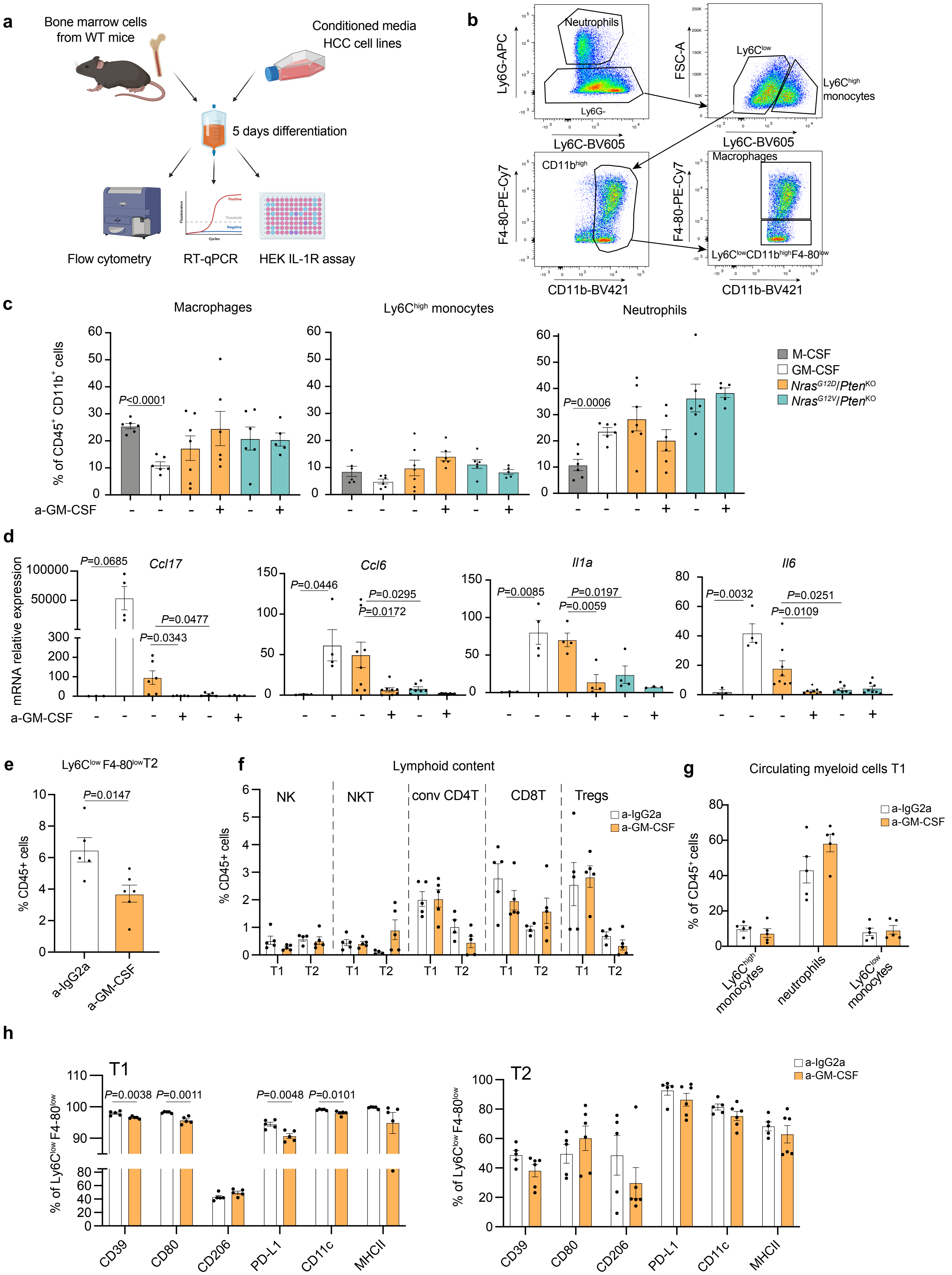

i

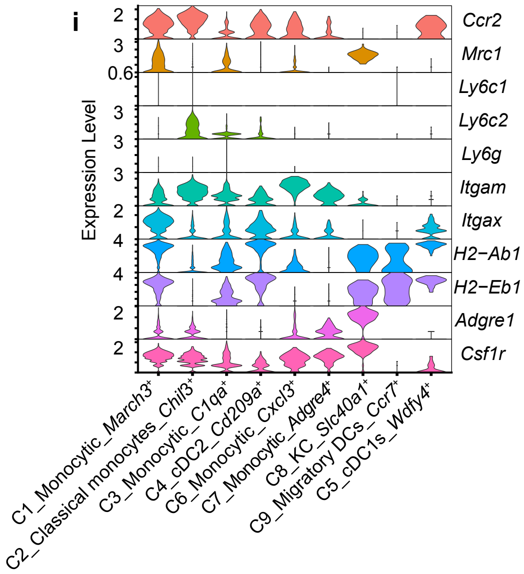
Expression Level
Ccr2
Mrc1
Ly6c1
Ly6c2
Ly6g
Itgam
Itgax
H2−Ab1
H2−Eb1
Adgre1
Csf1r
C1_Monocytic_March3+
C2_Classical monocytes_Chil3+
C3_Monocytic_C1qa+
C4_cDC2_Cd209a+
C6_Monocytic_Cxcl3+
C7_Monocytic_Adgre4+
C8_KC_Slc40a1+
C9_Migratory DCs_Ccr7+
C5_cDC1s_Wdfy4+

j

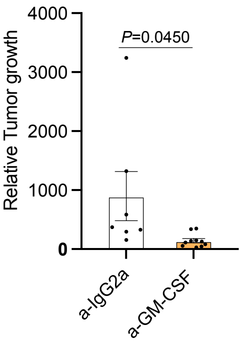
Relative Tumor growth
P=0.0450
a-IgG2a
a-GM-CSF

Supplementary Figure 7: GM-CSF inhibition hinders the differentiation of inflammatory Ly6C^{low} monocytes

a. Pipeline to differentiate bone marrow (BM) cells in conditioned media (CM) from HCC cell lines (see Method section) and follow-up procedures.

b. Flow cytometry gating strategy used to determine the abundance of depicted immune cells generated in BM differentiation experiments.

c. Percentage of macrophages, neutrophils and Ly6C^{high} monocytes relative to total myeloid cells obtained from BM cells differentiated in recombinant M-CSF (*n*=6), GM-CSF (*n*=6), or in CM prepared from distinct HCC cell lines, with or without GM-CSF neutralizing antibody (a-GM-CSF) (*Nras*^{G12D}/*Pten*^{KO} *n*=7, *Nras*^{G12D}/*Pten*^{KO} + a-GM-CSF *n*=6, *Nras*^{G12V}/*Pten*^{KO} *n*=6, *Nras*^{G12V}/*Pten*^{KO} + a-GM-CSF *n*=5).

d. mRNA expression of indicated genes in BM cells differentiated in recombinant M-CSF, GM-CSF, or in the CM of *Nras*^{G12D}/*Pten*^{KO} and *Nras*^{G12V}/*Pten*^{KO} HCC cell lines with or without a-GM-CSF (*Ccl17*: M-CSF *n*=3, GM-CSF *n*=4, *Nras*^{G12D}/*Pten*^{KO} *n*=6, *Nras*^{G12D}/*Pten*^{KO}+a-GM-CSF *n*=5, *Nras*^{G12V}/*Pten*^{KO} *n*=5, *Nras*^{G12V}/*Pten*^{KO}+a-GM-CSF *n*=5; *Ccl6*: M-CSF *n*=3, GM-CSF *n*=4, *Nras*^{G12D}/*Pten*^{KO} *n*=8, *Nras*^{G12D}/*Pten*^{KO}+a-GM-CSF *n*=8, *Nras*^{G12V}/*Pten*^{KO} *n*=7, *Nras*^{G12V}/*Pten*^{KO}+a-GM-CSF *n*=8; *Il1a*: M-CSF *n*=3, GM-CSF *n*=4, *Nras*^{G12D}/*Pten*^{KO} *n*=4, *Nras*^{G12D}/*Pten*^{KO}+a-GM-CSF *n*=4, *Nras*^{G12V}/*Pten*^{KO} *n*=4, *Nras*^{G12V}/*Pten*^{KO}+a-GM-CSF *n*=3; *Il6*: M-CSF *n*=3, GM-CSF *n*=4, *Nras*^{G12D}/*Pten*^{KO} *n*=8, *Nras*^{G12D}/*Pten*^{KO}+a-GM-CSF *n*=8, *Nras*^{G12V}/*Pten*^{KO} *n*=7, *Nras*^{G12V}/*Pten*^{KO}+a-GM-CSF *n*=8). Values represent fold change relative to one M-CSF BM sample, with *Ubc* as the housekeeping gene.

e-g. Abundance of intra-tumoral Ly6C^{low}F4/80^{low} cells (e), lymphoid cells (f) and circulating myeloid cells (g) from HDTVi-induced *Nras*^{G12D}/*Pten*^{KO}-bearing mice treated with a-IgG2a or a-GM-CSF at the indicated time points ((e) T2: a-IgG2a (*n*=5), a-GM-CSF (*n*=6); (f) T1: a-IgG2a (*n*=5), a-GM-CSF (*n*=5); (f) T2: a-IgG2a (*n*=4), a-GM-CSF (*n*=5); (g) T1: a-IgG2a (*n*=5) or a-GM-CSF (*n*=5).

h. Percentage of intra-tumoral Ly6C^{low}F4-80^{low} cells expressing the indicated phenotypic markers in HDTVi-induced *Nras*^{G12D}/*Pten*^{KO} HCC post treatment with a-IgG2a (*n*=5), a-GM-CSF (*n*=5) at T1, and a-IgG2a (*n*=5), a-GM-CSF (*n*=6) at T2.

i. Gene expression levels in control liver (*n*=1 mouse) and *Nras*^{G12D}/*Pten*^{KO} HCC (*n*=1 mouse) across the ‘Monocytic cell’ subpopulations.

j. Relative tumor growth of HDTVi-induced *Nras*^{G12D}/*Pten*^{KO} mice treated with a-IgG2a (*n*=7) or a-GM-CSF (*n*=10) after 4 weeks of treatment.

Graph shows mean±SEM (c-h, j). Statistical significance: unpaired two-sided Student's t-test in (c-e, h, j). See Supplementary Figure 7b and 9j-k for gating strategy (c,e-h). Source data are provided as a Source Data file.

Supplementary figure 8

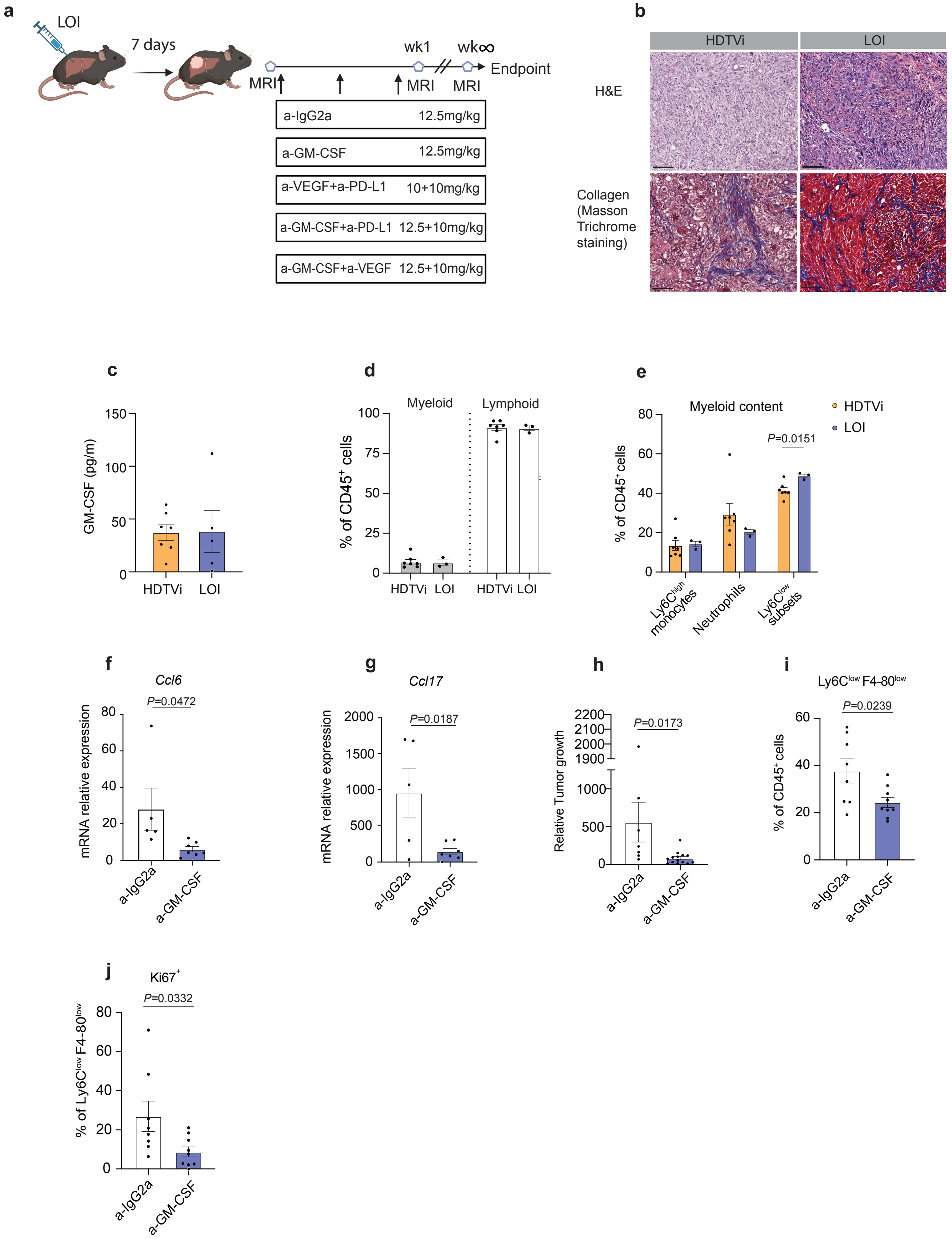

Supplementary Figure 8: *In vivo* GM-CSF and VEGF blockade abrogate the accumulation of pro-tumorigenic Ly6C^{low} myeloid cells in *Nras*^{G12D}/*Pten*^{KO}-driven HCC.

a. Experimental design: *Nras*^{G12D}/*Pten*^{KO} cells were orthotopically-injected into the liver (LOI) of C57BL6 mice. Seven days post-surgery, mice were monitored by weekly MRI and enrolled in the treatment groups upon tumor detection.

b. Representative H&E and Masson Trichrome staining performed on liver sections from HDTVi- and LOI-induced *Nras*^{G12D}/*Pten*^{KO}- HCC bearing mice at T2. Scale bars =100 µm; representative images from *n*=3 mice.

c. Quantification of intra-tumoral GM-CSF levels in end-stage HDTVi- (*n*=7) and LOI-induced (*n*=5) *Nras*^{G12D}/*Pten*^{KO} HCCs.

d. Myeloid and lymphoid cells quantification relative to total CD45^{+} leukocytes in end-stage HDTVi- (*n*=7) and LOI-induced (*n*=3) *Nras*^{G12D}/*Pten*^{KO} HCCs. Data include HDTVi-induced mice from Fig. 3a.

e. Percentage of intra-tumoral myeloid cell subsets relative to total CD45^{+} leukocytes in end-stage HDTVi- (*n*=7) and LOI-induced (*n*=3) *Nras*^{G12D}/*Pten*^{KO} HCC.

f-g. mRNA expression of *Ccl*6 (f) and *Ccl*17 (g) genes in end-stage LOI-induced *Nras*^{G12D}/*Pten*^{KO} HCCs treated with a-IgG2a (*n*=5) or a-GM-CSF (*n*=7). Values represent fold changes relative to one IgG2a-treated sample, with *Ubc* as the housekeeping gene.

h. Relative tumor growth of LOI-induced *Nras*^{G12D}/*Pten*^{KO} HCC treated with a-IgG2a (*n*=7) or a-GM-CSF (*n*=14) after two weeks of treatment.

i. Percentage of intra-tumoral Ly6C^{low}F4/80^{low} cells relative to total CD45^{+} leukocytes in end-stage LOI-induced *Nras*^{G12D}/*Pten*^{KO} HCCs treated with a-IgG2a (*n*=8) or a-GM-CSF (*n*=9).

j. Percentage of Ki67^{+}Ly6C^{low}F4/80^{low} cells in end-stage LOI-induced *Nras*^{G12D}/*Pten*^{KO} HCCs treated with a-IgG2a (*n*=8) or a-GM-CSF (*n*=9).

Graph shows mean±SEM (c-j). Statistical significance was determined by unpaired two-sided Student's t-test (e-j). See Supplementary Figure 9j-k for gating strategy (d-e,i-j). Source data are provided as a Source Data file.

Supplementary figure 9

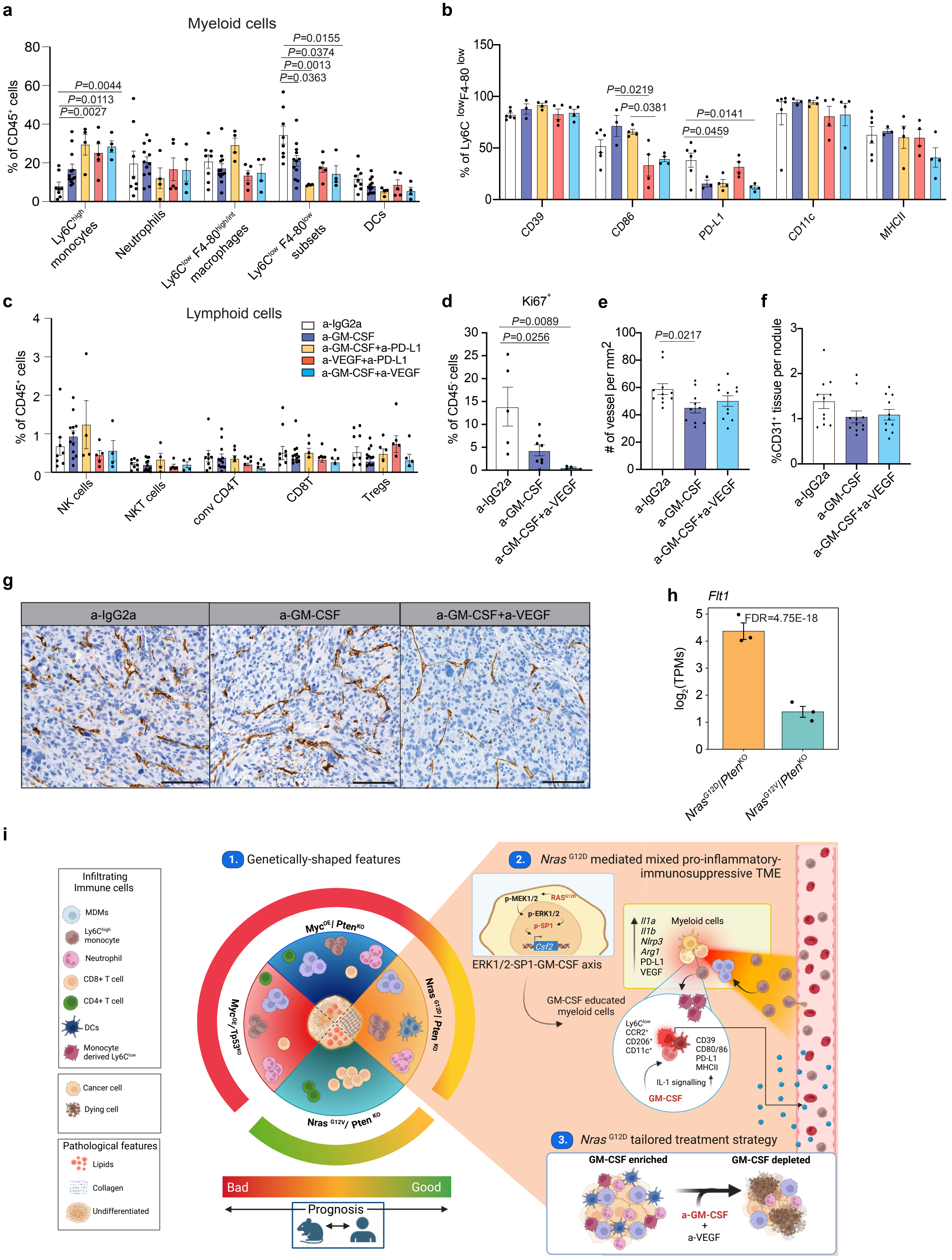

j

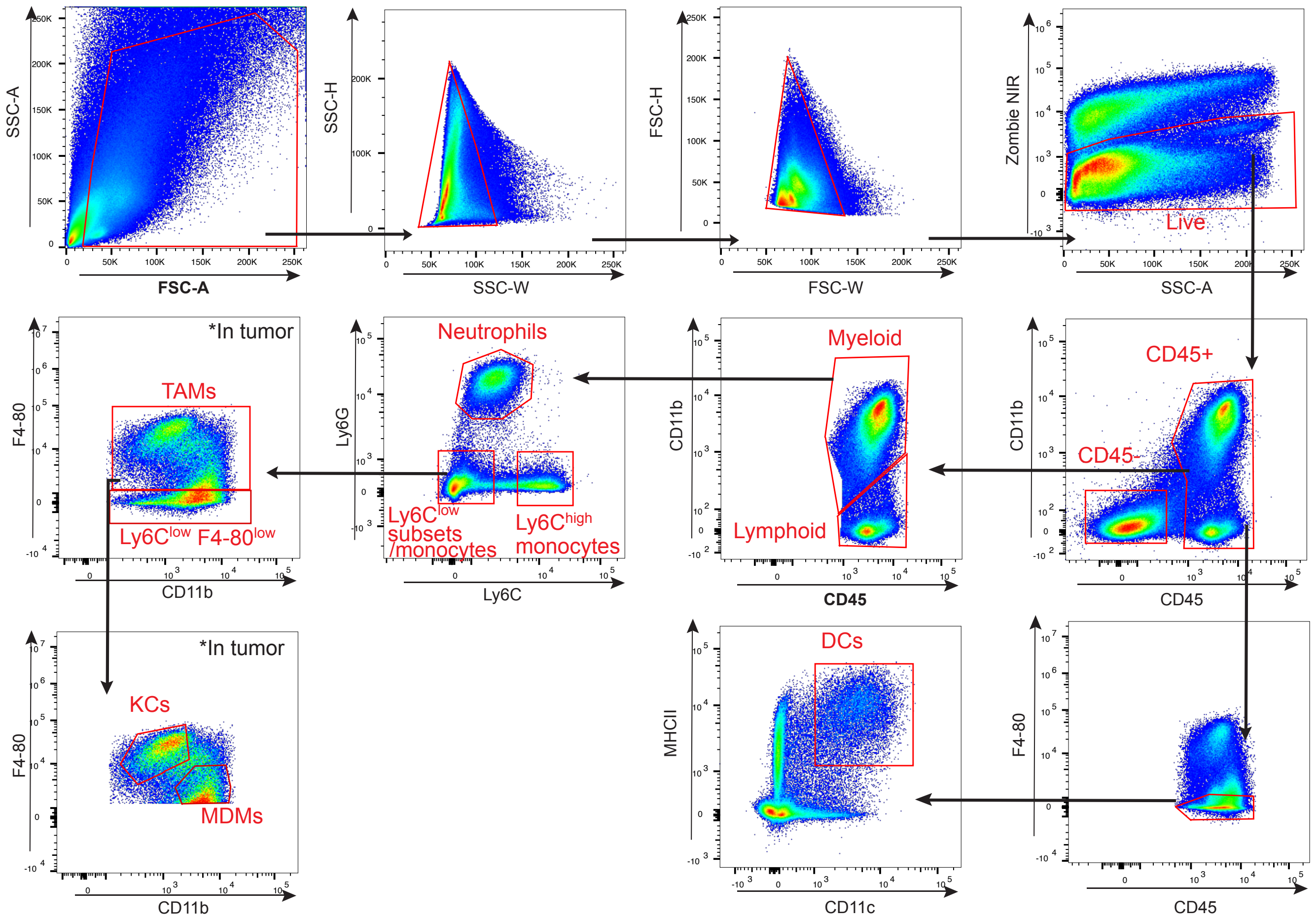

SSC-A
FSC-A
SSC-H
SSC-W
FSC-H
FSC-W
Zombie NIR
SSC-A
Live
*In tumor
F4-80
TAMs
Ly6Clow F4-80low
CD11b
Ly6G
Neutrophils
Ly6Clow subsets /monocytes
Ly6Chigh monocytes
Ly6C
CD11b
Myeloid
Lymphoid
CD45
CD11b
CD45+
CD45-
CD45
*In tumor
F4-80
KCs
MDMs
CD11b
MHCII
DCs
CD11c
F4-80
CD45

k

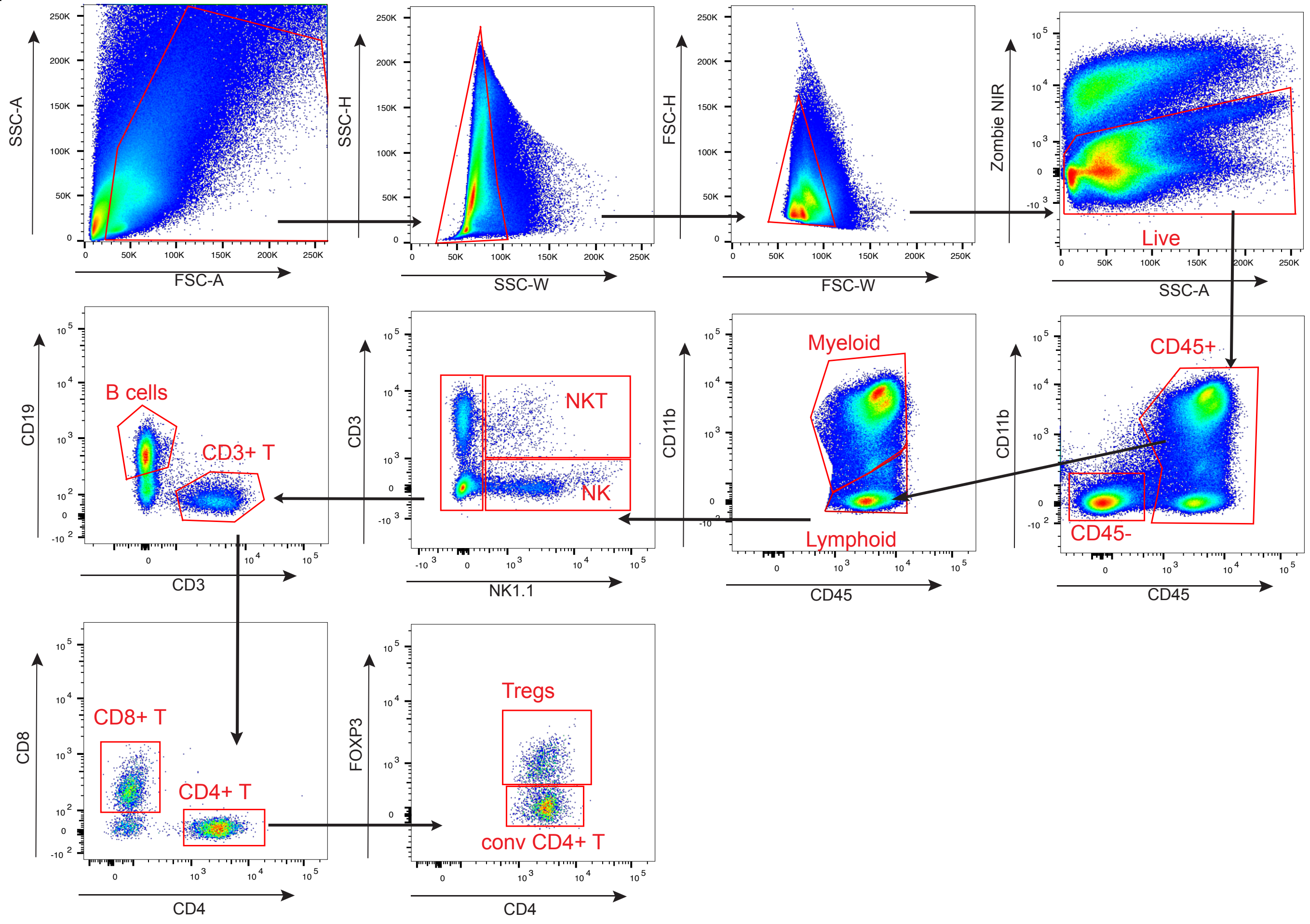

SSC-A
FSC-A
SSC-H
SSC-W
FSC-H
FSC-W
Zombie NIR
Live
SSC-A
CD19
B cells
CD3+ T
CD3
CD3
NKT
NK
NK1.1
CD11b
Myeloid
Lymphoid
CD45
CD11b
CD45+
CD45-
CD45
CD8
CD8+ T
CD4+ T
CD4
FOXP3
Tregs
conv CD4+ T
CD4

Supplementary Figure 9: Tumor microenvironment changes in *Nras*^{G12D}/*Pten*^{KO}-driven HCC upon treatment with immunomodulation strategies.

a. Percentage of intra-tumoral myeloid cell subsets relative to total CD45^{+} leukocytes in end-stage LOI-induced *Nras*^{G12D}/*Pten*^{KO} HCC treated with a-IgG2a (*n*=8), a-GM-CSF (*n*=12), a-GM-CSF+a-PD-L1 (*n*=4), a-VEGF + a-PD-L1 (*n*=5), and a-GM-CSF- + a-VEGF (*n*=4). Samples from (Supplementary Fig. 8i) included.

b. Percentage of Ly6C^{low}F4-80^{low} cells expressing the indicated phenotypic markers in end-stage LOI-induced *Nras*^{G12D}/*Pten*^{KO} HCC post treatment with a-IgG2a (*n*=6), a-GM-CSF (*n*=3), a-GM-CSF + a-PD-L1 (*n*=4), a-VEGF + a-PD-L1 (*n*=4), and a-GM-CSF + a-VEGF (*n*=4).

c. Percentage of adaptive immune cells relative to total CD45^{+} cells in end-stage LOI-induced *Nras*^{G12D}/*Pten*^{KO} HCC post treatment with a-IgG2a (*n*=8), a-GM-CSF (*n*=12), a-GM-CSF + a-PD-L1 (*n*=4), a-VEGF + a-PD-L1 (*n*=5), and a-GM-CSF + a-VEGF (*n*=4).

d. Percentage of CD45^{-}Ki67^{+} cells in end-stage LOI-induced *Nras*^{G12D}/*Pten*^{KO} HCC post treatment with a-IgG2a (*n*=5), a-GM-CSF (*n*=8) or a-GM-CSF and a-VEGF (*n*=4).

e-f Vessel counts per mm^{2} (e) and percentage of CD31 positive area (f) in LOI-induced *Nras*^{G12D}/*Pten*^{KO} end-stage HCC nodules post treatment with a-IgG2a (*n*=4), a-GM-CSF (*n*=4), and a-GM-CSF + a-VEGF (*n*=5).

g. Representative CD31^{+} IHC staining in LOI-induced *Nras*^{G12D}/*Pten*^{KO} endstage HCC nodules post treatment with a-IgG2a, a-GM-CSF or a-GM-CSF+a-VEGF (Scale bars = 1 mm; representative CD31^{+} for data shown in f).

h. *Flt1* mRNA expression in *Nras*^{G12D}/*Pten*^{KO} and *Nras*^{G12D}/*Pten*^{KO} cell lines (extracted from RNA-seq, *n*=3 for each cell line).

i. Graphical abstract.

j-k Representative flow cytometry plots depicting the gating strategy applied for sequential identification of myeloid cell (k) and lymphoid cell (l) populations in murine HCCs.

Graph shows mean±SEM (a-f, h). Statistical significance was determined by one-way ANOVA with Tukey's multiple comparison test (a-b,d) or by unpaired two-sided Student's t-test (e) and differential expression analyses (h). See Supplementary Figure 9j-k for gating strategy (a-d). Source data are provided as a Source Data file.

References

1 Nagtegaal, I. D. *et al.* The 2019 WHO classification of tumours of the digestive system. *Histopathology* **76**, 182-188 (2020). https://doi.org/10.1111/his.13975

2 Cancer Genome Atlas Research Network. Electronic address, w. b. e. & Cancer Genome Atlas Research, N. Comprehensive and Integrative Genomic Characterization of Hepatocellular Carcinoma. *Cell* **169**, 1327-1341 e1323 (2017). https://doi.org/10.1016/j.cell.2017.05.046

3 Wu, C. *et al.* Myeloid signature reveals immune contexture and predicts the prognosis of hepatocellular carcinoma. *J Clin Invest* **130**, 4679-4693 (2020). https://doi.org/10.1172/JCI135048

4 Xue, R. *et al.* Liver tumour immune microenvironment subtypes and neutrophil heterogeneity. *Nature* **612**, 141-147 (2022). https://doi.org/10.1038/s41586-022-05400-x

5 Croxford, A. L. *et al.* The Cytokine GM-CSF Drives the Inflammatory Signature of CCR2+ Monocytes and Licenses Autoimmunity. *Immunity* **43**, 502-514 (2015). https://doi.org/10.1016/j.immuni.2015.08.010
